# Aptamer-Functionalized Interface Nanopores Enable Amino Acid-Specific Peptide Detection

**DOI:** 10.1021/acsnano.3c10679

**Published:** 2024-02-14

**Authors:** Tilman Schlotter, Tom Kloter, Julian Hengsteler, Kyungae Yang, Lijian Zhan, Sujeni Ragavan, Haiying Hu, Xinyu Zhang, Jens Duru, János Vörös, Tomaso Zambelli, Nako Nakatsuka

**Affiliations:** † Laboratory of Biosensors and Bioelectronics, Institute for Biomedical Engineering, ETH Zürich, 8092 Zürich, Switzerland; ‡ Department of Medicine, Columbia University Irving Medical Center, New York, New York 10032, United States

**Keywords:** single-molecule sensing, force-controlled interface nanopore, fluid force microscopy, optical waveguide lightmode spectroscopy, DNA, phenylalanine

## Abstract

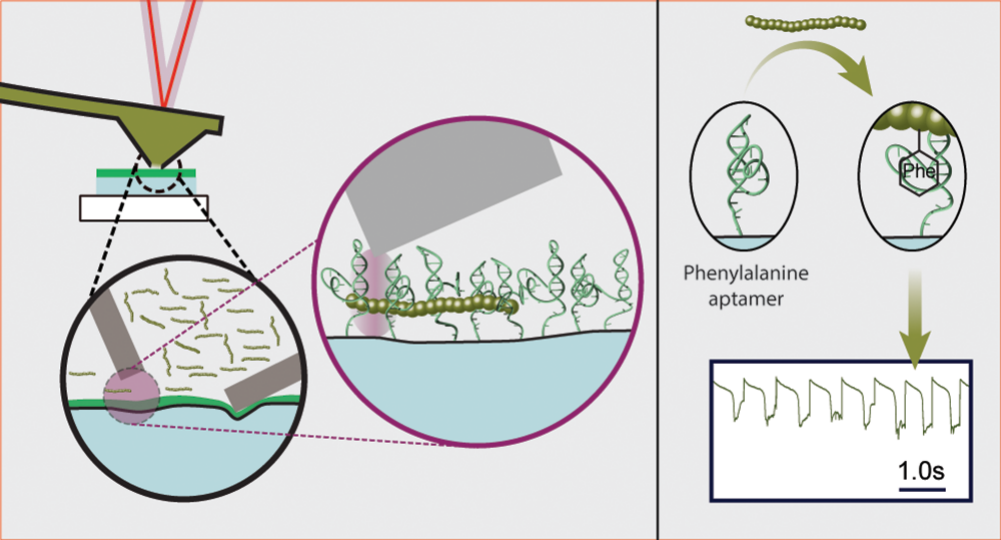

Single-molecule proteomics based on nanopore technology has made significant advances in recent years. However, to achieve nanopore sensing with single amino acid resolution, several bottlenecks must be tackled: controlling nanopore sizes with nanoscale precision and slowing molecular translocation events. Herein, we address these challenges by integrating amino acid-specific DNA aptamers into interface nanopores with dynamically tunable pore sizes. A phenylalanine aptamer was used as a proof-of-concept: aptamer recognition of phenylalanine moieties led to the retention of specific peptides, slowing translocation speeds. Importantly, while phenylalanine aptamers were isolated against the free amino acid, the aptamers were determined to recognize the combination of the benzyl or phenyl and the carbonyl group in the peptide backbone, enabling binding to specific phenylalanine-containing peptides. We decoupled specific binding between aptamers and phenylalanine-containing peptides from nonspecific interactions (e.g., electrostatics and hydrophobic interactions) using optical waveguide lightmode spectroscopy. Aptamer-modified interface nanopores differentiated peptides containing phenylalanine vs. control peptides with structurally similar amino acids (i.e., tyrosine and tryptophan). When the duration of aptamer–target interactions inside the nanopore were prolonged by lowering the applied voltage, discrete ionic current levels with repetitive motifs were observed. Such reoccurring signatures in the measured signal suggest that the proposed method has the possibility to resolve amino acid-specific aptamer recognition, a step toward single-molecule proteomics.

## Introduction

Cellular heterogeneity, which plays a critical role in disease states, necessitates the study of biological systems at single-cell resolution. Technological advancements in single-cell genomics (and the related fields of epigenomics and transcriptomics), as well as proteomics, are crucial to elucidate diverse cellular mechanisms and to tackle currently incurable diseases.^1^ While single-molecule oligonucleotide sequencing technologies have revolutionized genomics and are finding their way to clinical applications,^2^ advancements in single-cell proteomics remain limited.

Nonetheless, recent years have seen an emergence of developments in classical and modern proteomics technologies.^3^ For example, Edman degradation, a technique for identifying the amino acid sequence of a purified peptide, has been massively parallelized using chemically labeled peptide arrays (fluorosequencing).^4^ However, obtaining stable labels of multiple amino acids with a high degree of chemical specificity is nontrivial, and reagents used for peptide degradation often may elicit fluorophore destruction.^5^ While single-molecule protein fingerprinting has also been achieved (e.g., DNA-based point accumulation for imaging in nanoscale topography^6,7^ and digital enzyme-linked immunosorbent assays^8^), dependence on site-specific chemical labeling (that is not yet available for every amino acid^9^) leads to incomplete sequencing. A label-free approach and gold-standard method for protein identification is mass spectrometry (MS), which has seen significant advancement toward single-cell proteomics in the past decade.^10−13^ While the sensitivity of MS has been improved by advanced ion sources,^14^ bioinformatics,^15^ and other approaches,^16,17^ critical limitations remain in achieving high-throughput reads at the single-molecule level.

Nanopore technology has emerged in recent years as a powerful single-molecule stochastic sensor that not only enables nucleic acid sequencing,^18^ but also facilitates real-time *in situ* measurements of molecular interactions.^19,20^ However, the holy grail of single-molecule proteomics is not yet achieved due to the daunting demand of distinguishing not four, but 20 amino acids.^21^ While detection of one or few amino acids (e.g., cysteine and lysine) can be sufficient for protein fingerprinting,^22^ distinguishing amino acids of the same subgroup with identical chemical signatures, remains a grand challenge.^23^ Further, reading each amino acid at nanoscale intervals necessitates slow molecular translocation speeds to resolve the peptide sequence. Nanopore-induced phase-shift sequencing^24^ has been used to enzymatically ratchet and decelerate a DNA-peptide conjugate through a biological nanopore^25,26^ and eventually yield single-amino acid resolution.^27^ For solid-state nanopores, a key limitation is the nonspecific binding of intrinsically surface-active proteins with nanopore walls.^28−30^ By chemically modifying nanopore walls, the nonspecific interactions (e.g., electrostatics) can also be harnessed to facilitate specific interactions.^31−36^

In this work, we tackle such challenges by engineering chemically selective, dynamic solid-state nanopores featuring tunable orifices spanning 2–20 nm.^37,38^ To achieve peptide-specific stochastic sensing, nanopores were functionalized with DNA-based molecular recognition elements termed aptamers that serve three purposes: selective recognition of specific amino acids, deceleration of molecular translocation rates, and reduction of nonspecific binding to nanopore walls.^19,39^ As a proof-of-concept, we integrated a DNA aptamer validated prior for specific binding to phenylalanine (Phe)^40^ into dynamic nanopores. This approach enabled differentiation of peptides of identical length and charge solely based on the presence of the specific amino acid of interest (i.e., Phe). Selectivity vs. nonspecific amino acids with hydrophobic and aromatic side chains was demonstrated through discrimination of control peptide sequences with Phe residues replaced with tyrosine (Tyr) and tryptophan (Trp).

We correlated sequence-specific differences in peptide retention times during translocation to selective aptamer interactions to the Phe moiety, using optical waveguide lightmode spectroscopy (OWLS). This complementary measurement deconvolutes and quantifies specific aptamer-target binding interactions from electrostatic interactions between positively charged peptides and negatively charged DNA, and hydrophobic ring-stacking interactions between the phenyl group of aromatic amino acids and DNA bases. Inferring the specific sequence from the current signal is a challenging task, even in the case of DNA sequencing with nanopores.^41−44^ As a potential approach toward deducing peptide sequences with increased complexity, we extracted current levels using a simple changepoint detection algorithm to correlate reoccurring motifs of fixed length with an expected peptide signal generated from the known amino acid sequence. We employed Phe for the initial concept validation due to the availability of the aptamer. A recent report introducing aptamer sequences targeting various proteinogenic amino acids (arginine, glycine, glutamine, leucine, Tyr, and Trp) indicates the potential expansion of this method for future amino-acid specific proteomics analysis.^45^

## Results and Discussion

### Surface Chemistry and Characterization of Aptamer-Functionalized Interface Nanopores

**Figure 1:**
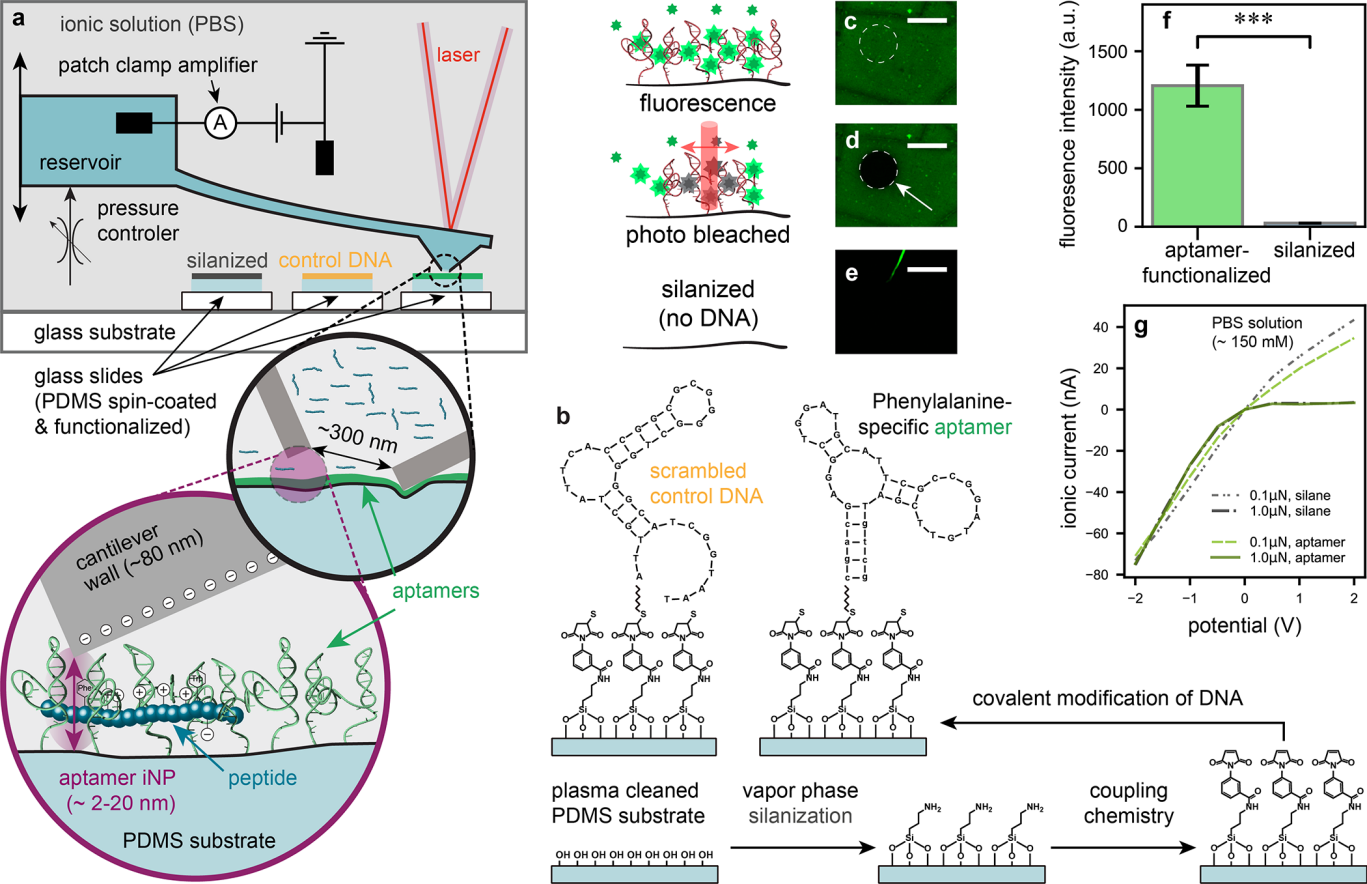
Aptamer functionalization of interface nanopores and validation of surface chemistry. (a) Schematic of the fluid force microscope setup, based on an atomic force microscope with a microchanneled cantilever mounted on the head-stage. The inside of the cantilever is connected to a pressure controller and an electrode. The same cantilever can be used to approach various functionalized glass slides such as negative controls (silanized, control DNA) and the specific aptamer-modified surface. (b) Schematic of the substrate functionalization steps, starting with a spin-coated and plasma cleaned polydimethylsiloxane (PDMS) surface, followed by chemical vapor phase deposition of aminosilanes, coupling chemistry, and subsequent covalent immobilization of thiolated DNA. The surface chemical modifications are schematized in an ideal manner. Schematic and corresponding fluorescence microscopy images of SYBR gold staining of (c) aptamer-functionalized, (d) photobleached, and (e) silanized PDMS substrates. Scale bars: 140 μm. (f) The fluorescence intensity of aptamer-functionalized substrates (*N* = 12) was statistically higher vs. silanized control substrates (*N* = 9). Error bars are standard errors of the means. Group means are significantly different *t*(19) = 20.26, *p* < 0.001. (g) Current–voltage curves of silanized vs. aptamer-functionalized substrates, measured at forces of 0.1 and 1.0 μN, show strong rectification post aptamer functionalization (*N* = 3).

For amino acid-specific peptide sensing with aptamer-modified nanopores, a force-controlled interface nanopore (iNP) setup^37,38^ that employs the fluid force microscope (FluidFM) technology^46^ was used. A nanopore is formed at the interface between a soft polydimethylsiloxane (PDMS) substrate and a hollow atomic force microscope (AFM) cantilever of the FluidFM made of silicon nitride (Figures 1a and S1). As the iNP system is fabricated out of solid-state materials, it remains robust under harsh conditions such as high ionic content, temperatures, or voltages that biological pores cannot withstand. The ionic current through an aperture of 300 nm at the apex of the cantilever tip is measured between two electrodes, one inside the cantilever reservoir and the other in the bulk solution, at a constant bias potential.

To achieve chemically modified iNPs, PDMS was spin-coated on glass slides and subsequently functionalized with either Phe-specific aptamers (binding affinity, *K*_d_ value of 16 μM vs. free Phe)^40^ or scrambled control DNA through sequential surface functionalization (Figure 1b). Considering the approximate surface area of single aminosilane molecules (∼3 Å^2^)^47^ relative to the size of aptamer 3-D conformations (on the order of tens of nm^2^),^48^ the free aptamer structure is the limiting factor for the surface density assembled on the surface of the PDMS. Covalent DNA modification of PDMS was confirmed using SYBR gold, a cyanine dye that exhibits >1000-fold fluorescence enhancement upon binding to DNA (Figure 1c). Photobleaching demonstrated that the fluorescence does not originate from background noise (Figure 1d). A control substrate with silanized PDMS with no subsequent DNA incubation (Figure 1e), showed negligible fluorescence intensity relative to the background (Figure 1f), validating DNA functionalization to specific substrates. Changes in contact angle on PDMS substrates were due to altered hydrophilicity with each functionalization step (Figure S2). Further, DNA surface functionalization inside the iNP was monitored by observing the current through the nanopore upon interfacing the cantilever with the PDMS. An increased ion current rectification^49^ is observed at both 0.1 μN and 1 μN when comparing the unmodified (silane) vs. aptamer-functionalized substrates due to the increased negative electric surface charge caused by the phosphate backbone (Figure 1g), confirming DNA immobilization inside the nanopore.

### Fluorescence Assays to Elucidate Aptamer Recognition to Phenylalanine in Peptide Sequences

**Figure 2:**
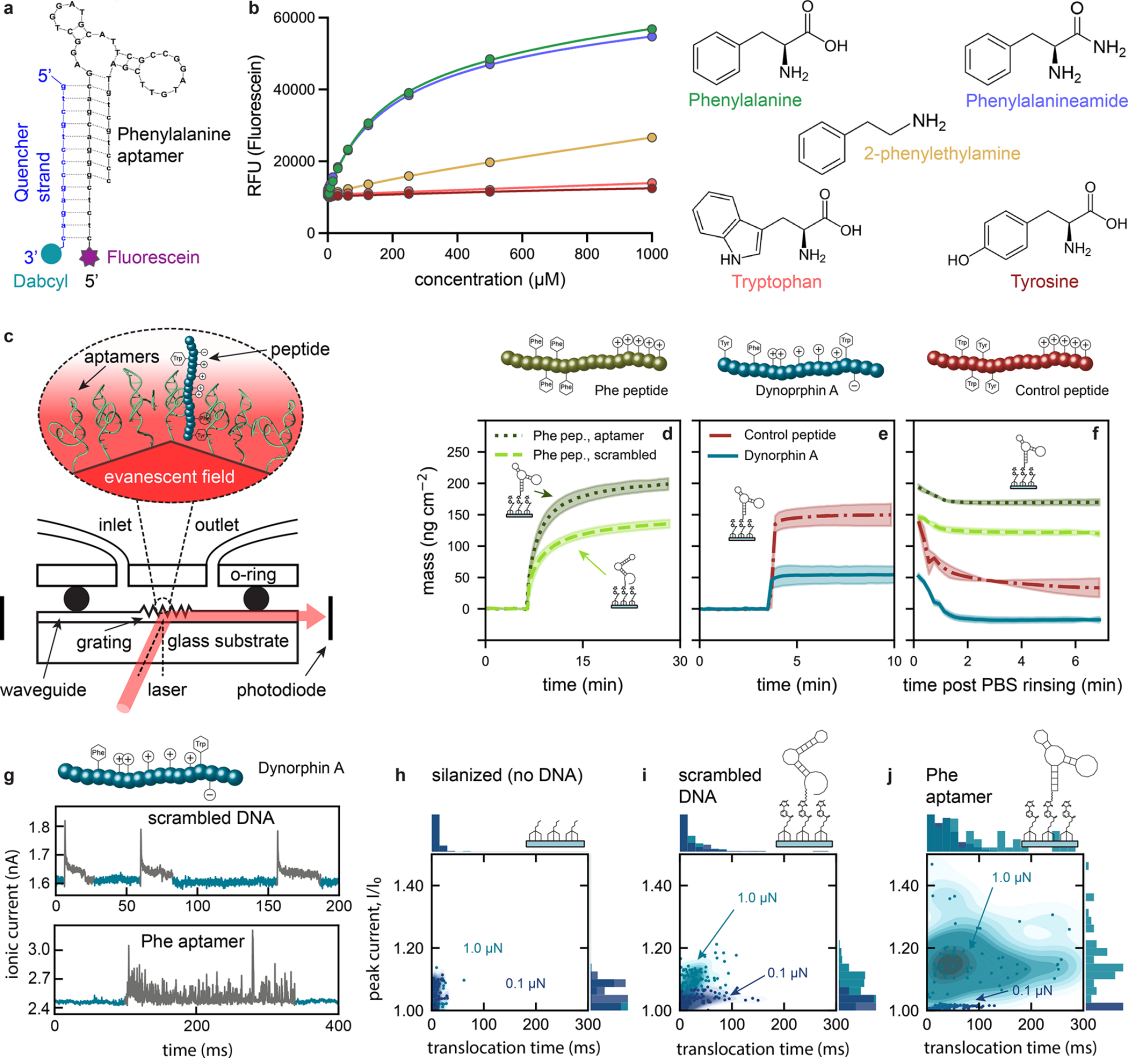
Fluorescence measurements for phenylalanine (Phe) aptamer recognition, optical waveguide lightmode spectroscopy (OWLS), and dynorphin A (Dyn) translocation measurements. (a) Phe aptamers modified with fluorescein on the 5′ end were incubated with a complementary quencher strand modified with dabcyl on the 3′ end. Upon target binding, the aptamers undergo a conformational rearrangement that leads to dissociation from the quencher strand, leading to increased fluorescence. (b) The relative fluorescence unit (RFU) of fluorescein was monitored in the presence of increasing concentrations of different molecules. Curves are the result of triplicate measurements, with standard deviations too small to be visualized. (c) Schematic of OWLS setup with close-up of the interface between the waveguide, aptamers, and peptides. (d) Mean adsorbed mass of the Phe peptide on waveguides functionalized with the Phe-aptamer (dotted dark green) and the scrambled control sequence (dashed green line). (e) Binding curves for aptamer-functionalized OWLS chips of Dyn (solid blue) and the control peptide (long dash-dotted red). (f) Unbinding curves of the different peptides upon rinsing with buffer. The shaded areas show the standard deviation from the mean (*N* = 3 different OWLS waveguides) and (d)–(f) share the same *y*-axis. (g) Current traces of Dyn translocations on scrambled DNA (top) and aptamer functionalized (bottom) surfaces. Density-scatter plots of translocations at 0.1 μN force (dark blue) and 1.0 μN (bright blue) through (h) silanized control substrates (*N*_0.1 μN_ = 62, *N*_1.0 μN_ = 80), (i) scrambled DNA (*N*_0.1 μN_ = 93, *N*_1.0 μN_ = 139), and (j) aptamer-functionalized (*N*_0.1 μN_ = 166, *N*_1.0 μN_ = 59) interface nanopores. All translocations were measured at an applied bias potential of 1.0 V. Density-scatter plots show the 0.05–0.95 percentile.

The Phe aptamer was originally isolated against free Phe in the original selection process.^40^ During isolation, counterselection was performed against Trp and Tyr to ensure selectivity vs. alternative hydrophobic amino acids. In this work, we used this Phe-specific aptamer to recognize this amino acid in the backbone of peptide sequences. To interrogate this binding mechanism, fluorescence assays were conducted where the Phe aptamer was modified with fluorescein on the 5′ end and hybridized to a complementary strand coupled to a quencher, dabycl, on the 3′ end (Figure 2a). When the aptamer recognizes a target, the conformational change that it undergoes for target binding releases the sequence from the hybrid strand, leading to an increase in fluorescence. Using this approach, different molecules with single displacement of functional groups compared to Phe were tested: phenylalanineamide and 2-phenylethylamine as well as chemically similar aromatic amino acids, Trp and Tyr (Figure 2b). The negligible binding to Trp and Tyr demonstrates the selectivity of the Phe-specific aptamer, and the focus of binding to the hydrophobic section of the molecule. The aptamer binds with comparable affinity to Phe and phenylalanineamide but with a weaker affinity to 2-phenylethylamine, which has a different preferred conformation. This, together with our peptide results, is consistent with recognition of the benzyl and the carbonyl groups, while not requiring the amino group. This ability to recognize the side chains within peptides of aptamers targeting amino acids, is a critical finding, that suggests that the detection of an expanded set of amino acid residues in peptides in parallel may be possible in the future.

### Optical Waveguide Lightmode Spectroscopy to Quantify Aptamer–Peptide Interactions

Interactions between the negatively charged DNA aptamers and positively charged peptides are governed by both electrostatic interactions and specific binding of Phe moieties with the Phe-specific aptamer. Further, the aromatic moieties of the peptides can lead to hydrophobic ring-stacking interactions with DNA bases. Thus, OWLS was used to extract the contribution of each of these phenomena. The OWLS system is based on a laser coupling into a waveguide via an optical grating, which enables *in situ* monitoring of biomolecular interactions in aqueous environments.^50^ Importantly, OWLS uses glass substrates; silane chemistry can be used in the same manner as the iNP measurements, enabling direct comparison between the two methodologies. As shown in Figure 2c, OWLS quantifies the mass of biomolecules that binds to the DNA-functionalized surface by precisely measuring the incoupling angles of the transverse electric and magnetic modes using photodiodes.

The interactions of the aptamer with three different peptides with varying charges and number of Phe motifs were investigated by OWLS: the natural opioid neuropeptide, Dynorphin A (Dyn),^37^ with four positive net charges at physiological pH and one Phe moiety, and two peptides designed with specific sequences: Phe peptide with five positive charges and four Phe moieties and Control, a peptide with the same sequence except with each of the four Phe replaced by either Tyr or Trp (Tables S1 and S2). This negative control was designed such that the peptide has the same charge and similar chemical signature as the Phe peptide (aromatic, hydrophobic).

For the Phe peptide interacting with the Phe aptamer-modified substrate, we observed an absorbed mass of 198 ± 9 ng cm^–2^ (Figure 2d). The adsorbed mass is lower (140 ± 7 ng cm^–2^) when the Phe peptide is exposed to a surface coated with control DNA, in which the same number and type of nucleotides are retained from the specific Phe aptamer but reordered to alter the molecular recognition. As the scrambled control DNA has the same charges as the specific aptamer while lacking specific binding sites, the adsorbed mass of the Phe peptide is due to nonspecific, electrostatic interactions. The statistically significant difference in adsorbed mass on the specific Phe aptamer vs. scrambled DNA (Figure S4) is attributed to specific aptamer recognition of the Phe peptide. The extent of nonspecific binding of the Control peptide on the aptamer-modified substrate is comparable (149 ± 17 ng cm^–2^), due to electrostatic interactions between the control DNA surface and the Phe peptide (Figure 2e). The Dyn, which has one less positive charge and only one Phe vs. the four Phe moieties of Phe peptide, shows an ∼4-fold lower mass adsorption (52 ± 14 ng cm^–2^), indicating the influence of the presence of specific amino acid groups. The ∼3-fold lower binding of Dyn to the specific aptamers in contrast to the electrostatic interactions in control DNA/peptide systems, is likely due to the charge distribution along the peptide backbone.^51^ This distribution alters the density of potential electrostatic interactions for the Phe and Control peptides (that localize five positive charges at the C-terminus) vs. Dyn, where positive charges are spread throughout the peptide length. Figure S5 illustrates the correlations between the interaction density and assembled peptide height on the aptamer-modified surface.

Further, the binding kinetics vary between the three peptides on either aptamer-functionalized or scrambled DNA. While the Phe peptide takes ∼30 min to reach equilibrium upon exposure to the Phe aptamer-modified substrates, the Control and Dyn binding occurs on the order of a few minutes. The slower binding kinetics of the Phe peptide is likely due to the added contribution of molecular recognition of Phe vs. solely electrostatic interactions driven by charged moieties in the Control and Dyn samples. The unbinding kinetics of the peptides were extracted by rinsing with buffer after signal saturation (Figure 2f). The Control and Dyn showed fast and almost full unbinding from the aptamer-modified surface, while the Phe peptide was retained on the surface despite rinsing. Retention of the Phe peptide even on the scrambled nonspecific DNA surface, can be explained by the reported affinity of Phe for DNA bases due to extensive ring-stacking interactions, which is not observed for Trp and Tyr.^52^ The maximal binding and retention observed for the Phe peptide on the specific aptamer-modified surface results from the combined effect of specific (aptamer-Phe binding) and nonspecific (electrostatic, hydrophobic) interactions. This effect predicted increased retention times for specific peptides inside aptamer-functionalized iNPs.

### Differentiation of Electrostatic vs. Amino Acid-Specific Interactions for Dynorphin A

An advantage of the iNP system with a mobile nanopore is the ability to test different surface functionalizations using the same cantilever, enabling direct comparisons between measurements. In each experiment, specific Phe-aptamer interactions were interrogated in parallel with control measurements from silanized surfaces (no DNA) and surfaces functionalized with scrambled DNA. As high salt concentrations of 0.5 M KCl resulted in unbinding of the peptides from the aptamers (see Figure 2f), a physiological concentration of ∼150 mM salt conditions (PBS) was used for all translocation measurements reported herein. Ionic currents for Dyn translocation events on scrambled vs. Phe aptamer-functionalized iNPs are shown in Figure 2g (longer traces are shown in Figure S6). While translocations on the scrambled aptamer yield defined short peaks with translocation times below 100 ms (mean and standard deviation: 26 ± 28 ms), translocations on aptamer-modified substrates show longer retention times up to 300 ms (70 ± 124 ms), indicative of sequence-specific interactions.

The influence of applied force on the substrate (which correlates to the pore size) on the aptamer–peptide interactive events was studied. An in-depth characterization of the nanopore size with respect to the applied force was conducted in prior work.^37^ Reducing the pore size (at a constant voltage bias of 1.0 V) by increasing the applied force from 0.1 to 1.0 μN, corresponds to a reduction in estimated pore size from *ca*. 10 to 5 nm respectively, yielding a smaller sensing volume and an increased occupied space fraction of the biomolecules. The estimated pore size includes the passivation layer and assembled aptamers, which means that the effective pore size is even smaller. While the relative peak current (defined as the maximum current of a translocation event divided by the baseline current) increased moderately with the higher applied force for the silanized (no DNA) substrates (Figure 2h), this effect was amplified for surfaces modified with DNA: both the scrambled control (Figure 2i) and the Phe-specific aptamers (Figure 2j). Confinement due to a higher applied force increases the probability of electrostatic interactions between the positively charged peptides and negative DNA surfaces, while positively charged silane surfaces repel the peptides. When comparing the Dyn translocation time through aptamer-functionalized vs. scrambled DNA iNPs, the retention times inside the pores are approximately twice as long due to the combined effect of molecular binding events and electrostatics. This result demonstrated that sufficiently small pore sizes (<5 nm) are necessary to observe specific interactions between the aptamer and the Phe in the peptide backbone. Therefore, subsequent nanopore measurements were conducted with an applied force of 1.0 μN.

### Translocation Time Dependency on Amino Acid-Presence Due to Specific Interactions

**Figure 3:**
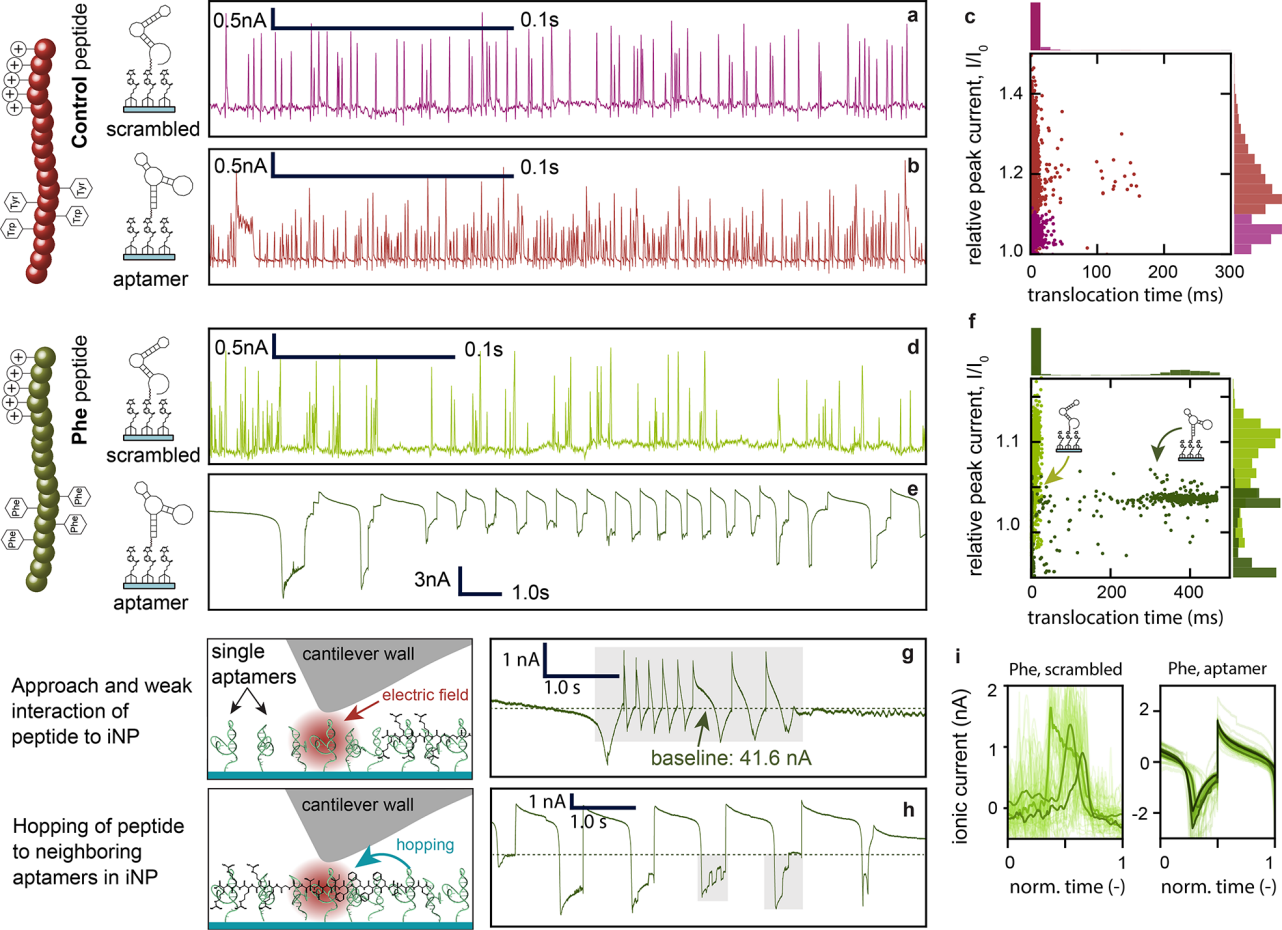
Translocations and shape analysis of Control vs. phenylalanine (Phe) peptides on aptamer vs. control substrates. All measurements were conducted at a bias potential of 1.5 V. Time scales vary for certain comparisons to enable the visualization of different current shapes. Ionic current traces of the Control peptide interactions through (a) scrambled DNA and (b) aptamer-modified interface nanopores (iNPs). (c) Scatter plot of Control peptide translocations classified as spikes on scrambled DNA (*N* = 1180) and aptamer-modified substrates (*N* = 4574, 0.1–0.9 percentile). Current traces of Phe peptide through (d) scrambled DNA and (e) aptamer-functionalized iNPs. (f) Scatter plot of Phe peptide translocations classified as spikes on scrambled DNA (*N* = 341) and aptamer-modified substrates (*N* = 553, 0.1–0.9 percentile). (g) and (h) show a shorter current trace of the signal in (e) with (g) weak peptide–pore interactions and fast translocations and (h) strong interactions leading to the hopping of peptides within the pore sensitive region. (i) Mean shape of the OPTICS-UMAP clustering of Phe-peptide translocations on a scrambled (left) and an aptamer-functionalized (right) iNP. The first 3 clusters are shown.

The ionic currents and translocation times of the Control (Figure 3a–c) vs. Phe (Figure 3d–f) peptides were measured on both scrambled DNA and Phe aptamer-functionalized substrates. The individual peaks were detected and analyzed using a continuous wavelet transformation of the current signal, as described in Figures S7 and S8. Filtered (Figure S9) ionic currents of the Control peptide through both scrambled (Figure 3a) and aptamer-modified (Figure 3b) iNPs at an applied potential of 1.5 V showed translocation times of 15 ± 43 and 21 ± 64 ms, respectively (Figure 3c). Unchanged translocation times and translocation frequencies (Figure S10) of the Control peptide regardless of specific vs. nonspecific iNPs, confirmed that the minimal retention times are governed solely by electrostatics, corroborating the results observed in OWLS. Translocation shapes for the control measurements (Figure S11) can be classified as sharp peaks, which indicate low nonspecific peptide–nanopore wall interactions.^48^ Some translocations show a small drop before the sharp current increase, which has been previously observed^38^ and may be attributed to the peptide orientation inside the nanopore as the positive charges are on the C-terminal side of the peptides.

Filtered ionic currents of the Phe peptide through scrambled DNA-modified iNPs at an applied potential of 1.5 V showed fast translocation times <20 ms (11 ± 11 ms, Figure 3d). Alternatively, with the same applied potential, longer translocation times of >400 ms (190 ± 234 ms) were observed for Phe peptides translocating aptamer-modified iNPs (Figure 3e). Increased retention times through aptamer-modified vs. control DNA iNPs are due to specific interactions of Phe amino acids in the peptide backbone with the aptamers (Figure 3f). Fast translocations that led to continuous transient currents are attributed to weak or minimal interactions, leading to repetitive translocation peaks (Figure 3g, shaded area). Binding and unbinding of peptides with aptamers within the sensitive iNP region led to steep current changes with longer translocation times (Figure 3h, shaded areas).

To understand the translocation dynamics, the single events were normalized in length and shape analysis was conducted using the UMAP^53^ dimension reduction algorithm followed by a density-based clustering (11 clusters were identified) via the DBSCAN algorithm.^54^ The time-normalized ionic current signals are then overlaid (Figure 3i). After an initial current drop, a steep current increase followed by a slow current decay was seen in the dimension reduction cluster mean shapes for the specific aptamer-peptide combination (right subplot), while for scrambled DNA substrates, short peaks were identified (left subplot). Further analysis of the peak analysis and clustering for the specific (Figure S12) and nonspecific (Figure S13) interactions are detailed in the Supporting Information.

### Low Voltage Recordings Resolve Peptide–Aptamer Interactions in the Nanopore

**Figure 4:**
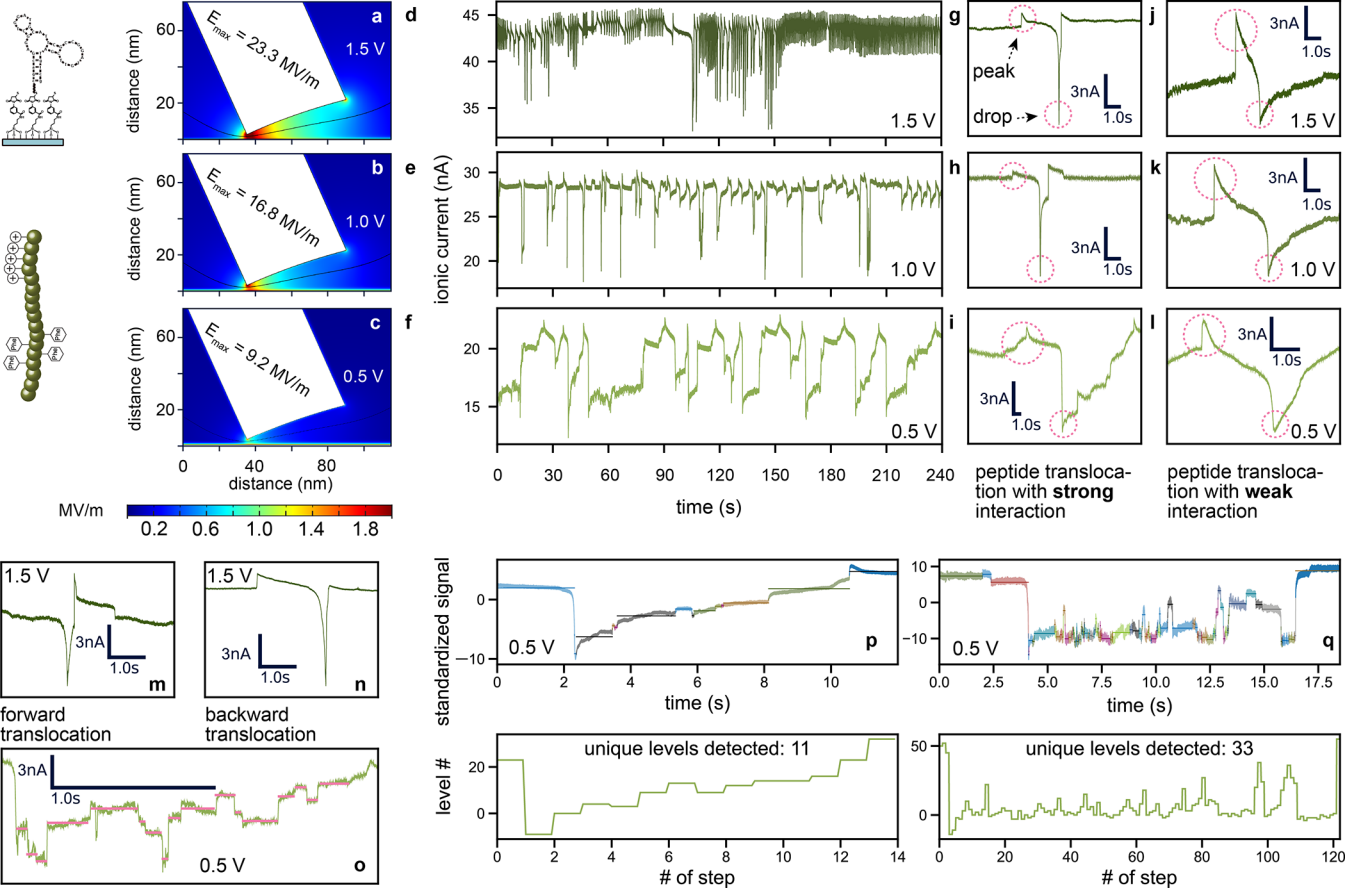
Lower bias potentials (0.5 V) lead to increased specific aptamer–phenylalanine (Phe) interactions. COMSOL simulations of the electric field distribution inside the interface nanopore (iNP) with surface charge (details in SI, section 3.5) at potentials of (a) 1.5, (b) 1.0, and (c) 0.5 V, with corresponding maximum values along the black streamline. Filtered and down-sampled ionic current traces of Phe-peptide translocations through an aptamer-functionalized nanopore at (d) 1.5, (e) 1.0, and (f) 0.5 V. Single translocations at distinct potentials with (g–i) strong interactions and (j–l) weaker interactions. Current traces that may correlate to (m) forward and (n) backward translocation of a peptide. (o) Peptide translocation with 17 distinct current levels at a potential of 0.5 V. (p) Short translocations of about 8 s duration with respective steps and current levels detected at 0.5 V. (q) Longer translocation of about 15 s duration with respective steps and current levels detected at 0.5 V.

Reducing the applied potential between the electrodes from 1.5 to 0.5 V, decreases the electric field (*E⃗*) inside the iNP (Figure 4a–c). The field confinement defines the sensitivity zone (Figure S15) where molecular translocation leads to changes in the measured current. Following *F⃗* = *qE⃗*, where *q* is the net charge of the molecule (+5e), when the voltage is reduced, the electrostatic force driving the molecule through the pore (*F⃗*) is also reduced, while aptamer–peptide interactions remain conserved. Thus, a reduction in applied voltage (0.5 V vs. 1.5 V) manifests as an increase in the retention time (several seconds vs. ms) of the Phe peptide through the aptamer-modified iNP (Figure 4d–f). The Phe peptide translocation events often show a rapid, short current increase due to excess charge being brought to the nanopore by the charged peptide in the form of counterions.^37,38^ Then, a slow current decay is observed when the peptide is adopting a favorable conformation for translocation based on the electric field that orients charged moieties (*C*- to *N*-terminus direction).^55^ The entry of the peptide into the nanopore leads to a current blockade upon specific binding with aptamers. The subsequent increase in current back to the baseline occurs when the peptide unbinds from the aptamers and exits the nanopore. This process takes longer if the bias potential is decreased (0.5 V, Figure 4g–l) because the peptide remains bound to the aptamer for a longer period with a weaker electrostatic pull. Alternatively, if the peptide translocates from the opposite direction (*N*- vs *C*-terminus) through the iNP, a current drop is first observed, followed by a current peak (Figure 4m,n). The influence of the peptide orientation on the measured current signals correlates with simulated translocation peak shapes (Figure S16).

At lower potentials (0.5 V), the rebinding events of the Phe moieties in the peptide backbone to neighboring aptamers in the nanopore led to signals with discrete current levels that may be correlated to specific peptide residues (Figure 4o). However, translocation events with fewer steps (Figure 4p) and many more steps (Figure 4q) were also detected, suggesting that these current levels originate from peptide hopping within the nanopore, which likely arises due to (1) the pore geometry that extends perpendicular to the translocation direction for tens of nanometers and therefore provides additional binding positions and (2) the presence of four Phe moieties in the backbone further increasing the possible number of rebinding events. To interrogate whether the observed current levels can be correlated to the specific peptide sequence, a motif search based on the autocorrelation of a sliding window was performed (SI, section S4.7). A virtual signal based on the peptide sequence was generated and compared with the most reoccurring motif above a certain correlation threshold. While correlations between the peptide sequence and the experimental signal were found, the demonstration that the correlation is causal requires further investigations outside the scope of this work.

## Conclusion

We have harnessed aptamers to create a stochastic sensor that enables the amino-acid-specific detection of peptides. Explicitly, Phe-aptamer-modified iNPs differentiated peptides with the same charge and residue number but with different numbers of Phe motifs. The generalizable surface chemistry to couple DNA sequences covalently to iNPs was validated both by fluorescence microscopy (dye-stained DNA) and by ion current rectification measurements. Further, using both OWLS and nanopore-based measurements, we have verified that the Phe-specific aptamers recognize not only single Phe as reported prior,^40^ but also Phe motifs in the peptide backbone. Using fluorescence assays, we also deduced the binding mechanism of the aptamer to Phe motifs in peptide sequences, which occurs via fragment-based recognition of the combination of the benzyl and carbonyl groups. A significant advantage of our approach is the generalizability and adaptability; different amino acid-specific aptamers can be integrated using the same surface modification strategy into on-demand, size-tunable, and mobile nanopores. To date, we have been limited by the availability of high-affinity and selective aptamers for amino acids. Yet, with improved selection strategies for small-molecule aptamers, such sequences are already in the pipeline.^56^

When predicting the approximate height of Phe aptamers based on their most thermodynamically stable conformation (modeled by *MFold*(57)) under the environmental conditions tested, the aptamers are ∼5 nm. Thus, the pore is “pre-clogged” with DNA, ensuring peptides interact with the aptamers when pulled through the pore by applying a large enough potential bias. A challenge we foresee is the detection of minimally charged peptides or unknown sequences, where we cannot predict the necessary applied voltage to drive the peptide through the pore electrostatically. Nevertheless, combining this technology with other techniques such as gel electrophoresis that discriminates peptides based on charge and mass would expand existing capabilities of peptide differentiation.

An important advantage of this methodology is the ability to cross-reference various substrates functionalized with different chemistries using the same cantilever. When conducting measurements with solid-state nanopores on the order of 5 nm, variability in pore size and shape results in challenges in reproducibility and referencing vs. controls. In this reported system, measurements through specific vs. nonspecific (e.g., modified with scrambled DNA) iNPs can be compared directly, reducing the experimental variation. Thus, in addition to providing an innovative approach for amino-acid-specific stochastic sensing, aptamer-functionalized iNPs facilitate multiplexed readouts of surface binding interactions with different surface modifications and analytes within one experiment. While full peptide sequencing has not yet been achieved, our findings suggest that peptides with high degrees of chemical and structural similarity can be resolved selectively. Moreover, the integration of serial nanopores into the current technology, with distinct amino acid-specific aptamers functionalized at consecutive pores, may actualize a platform for protein sequencing.

## Experimental Section

### Experimental Setup

The experimental setup of the force controlled nanopore is based on the FluidFM technology,^46^ which combines an AFM and a microchanneled cantilever with an apex at the tip. The cantilever (Cytosurge, nanopipette) is made from Si_3_N_4_ and has a 10 × 10 × 7 μm^3^ pyramid at the extremity with an aperture of 300 nm diameter at its apex and a nominal spring constant of 2 N/m. The iNP is formed between a soft PDMS substrate and the microchanneled AFM cantilever, which is mounted on a scanning ion-conductance microscopy setup with a mounting angle of α = 11° as previously reported.^58^ A controller (Nanosurf, C3000) modulates the force between the cantilever tip and the PDMS substrate. On the AFM head (Nanosurf, Flex-Bio) a patch clamp amplifier (Tecella, Pico) is mounted, which connects the reservoir electrode to the amplifier and measures the ionic current between the reservoir and the reference electrodes. A Faraday ring is mounted on the AFM stage to insulate the setup from electromagnetic radiation while also fixing the sample dish. The cantilever is mounted on a holder that had a reservoir on the back side. For the calibration of the spring constant, the frequency spectrum and resonance peak of each used cantilever is measured (Sader method^59^). The ionic current through the apex is measured by two silver chloride (Ag/AgCl) quasi-reference electrodes. Measurements with similar baseline currents at the same potential are compared with each other. The sampling rate for all measurements was 40 kHz. A more detailed setup description including cantilever and pore geometries can be found in the Supporting Information of our previous work.^37^ Functionalized substrates were used for a maximum period of 2 weeks.

### Treatment of Cantilevers and Teflon Holder

The *N*-2-hydroxyethylpiperazine-*N*-2-ethanesulfonic acid (HEPES) buffer was purchased from ThermoFisher Scientific at 1 M concentration and was diluted to 20 mM using ultrapure water (18.4 MΩ·cm, Milli-Q gradient A 10 from Merck-MilliPore). A 0.1 mg/mL PAcrAm-PMOXA (PMOXA, SuSoS, Switzerland) coating solution was prepared in the diluted HEPES buffer. Pores were plasma cleaned for 1 min (air plasma, at 18 W, using a PDC-32G; Harrick Plasma Cleaner) and after the cleaning, 10 μL of the PMOXA solution was added into the cantilever reservoir. From the reservoir, the solution was pushed through the microchannel using a pneumatic connector and the pressure control unit of the FluidFM setup. Then, the pore was submersed in the PMOXA solution for 90 min then rinsed two times with MQ water prior to use. Finally, the reservoir connector with the electrode and the connection to the pressure control was mounted on the backside reservoir of the probe and subsequently sealed with paraffin wax heated to 90 °C.

To passivate the Teflon reservoir, a 3 mg/mL solution of bovine serum albumin (BSA, Sigma-Aldrich) in 150 mM phosphate-buffered saline (PBS, pH 7.4, Thermo Fisher, Gibco, 10010015) was incubated on the reservoir surface for 15 min. The reservoir was then rinsed twice with PBS. A control measurement to check that the BSA itself does not generate a signal after treating the Teflon block is provided in Figure S3. For this control experiment, the Teflon reservoir was filled with pure PBS after BSA treatment and then the solution was aspired into the cantilever and subsequently measured using the same protocol as typical experiments. Without passivation, the peptide in the solution would stick to the Teflon surface, significantly reducing the peptide concentration and hindering observations of translocation events during measurements.

### Fabrication of the PDMS Substrates

The soft substrate is fabricated by spin-coating PDMS (Sylgard 184) at a 1:10 curing agent ratio on a round microscope cover glass (diameter 24 mm, thickness 0.13–0.16 mm, Thermo Fisher). Before curing, PDMS is left at room temperature for 5 min to achieve a homogeneous surface. Then, the PDMS is cured in the oven at 80 °C or on a hot plate at 210 °C for 120 min. The cured PDMS layer on glass was then glued into a dish chamber (Willco Wells). Prior to surface functionalization with DNA, the PDMS-coated slides were submerged for 30 min in fresh *n*-hexane, which was repeated three times to remove excess uncured residues. After the hexane solution was fully evaporated, the surface was rinsed with MQ water, blown dry with nitrogen, and plasma cleaned for 2 min at 200 W.

### DNA Functionalization

All substrates (PDMS or OWLS chips) were functionalized with DNA sequences (Phe aptamers, or scrambled control sequences, Table 1) using a previously reported protocol.^47^ Briefly, (3-aminopropyl)trimethoxysilane (APTMS) was vapor deposited on PDMS substrates at 40 °C for 1 h. To cross-link amine-terminated silanes to thiolated DNA, 1 mM 3-maleimidobenzoic acid *N*-hydroxysuccinimide ester (MBS) was dissolved in a 1:9 (v/v) mixture of dimethyl sulfoxide and 1× PBS and incubated for 1 h. Concurrently, the DNA was prepared for coupling by reducing the disulfide bonds using 50-fold excess tris(2-carboxyethyl) phosphine (TCEP) relative to DNA concentration for 1 h. The aptamer solution was then diluted to 5 mM in 1× PBS and purified with Zeba spin desalting columns (7K MWCO, 0.5 mL, Thermo Fisher Scientific AG, Reinach, Switzerland). The DNA was denatured at 95 °C for 5 min then cooled to room temperature prior to surface attachment.

**Table 1:** Sequences of Phenylalanine-Specific Aptamer and the Scrambled Sequence Used in This Work

sequence name	nucleic acid sequence
phenylalanine aptamer	5′-CGACGAGGCTGGATGCATTCGCCGGATGTTCGATGTCG-3′
scrambled sequence	5′-ATTGCTATTCACCGGCGCGGGGCTGGGGCATCGGTAAT-3′

### Fluorescence Verification and Quantification of DNA Functionalization

SYBR gold nucleic acid gel stain (Invitrogen, Carlsbad, CA, U.S.A.) was used to stain and visualize DNA on the functionalized slides. The SYBR gold dye was diluted 8000-fold into 1× Tris-EDTA buffer to yield the staining solution. Slides were immersed in this staining solution for 20 min at room temperature in the dark. After two rounds of rinsing with MQ water, the fluorescence was measured by using a confocal laser scanning microscope. The dye was excited at a wavelength of 488 nm. Fluorescence quantification from the acquired fluorescence images was conducted using the software FIJI. Mean gray values have been quantified across the area of the pictures as mean fluorescent intensity. Those mean fluorescence values were averaged over the different pictures to yield mean values.

### Aptamer Fluorescence Assays

The assay procedure was followed as reported prior.^45^ To measure the aptamer response to the target analytes through the strand displacement reaction, the quenching ratio was determined between FAM-labeled phenylalanine aptamer (/56-FAM/CTC TCG GGA CGA CGA GGC TGG ATG CAT TCG CCG GAT GTT CGA TGT CGT CCC) and the corresponding dabcyl-labeled quencher strand (GTC GTC CCG AGA G/3 Dab/). The aptamer and quencher strands were mixed at a predetermined ratio (50 nM: 150 nM), placed in boiling water for 5 min, and allowed to cool to room temperature. Dilutions of the target solution were mixed with an equal volume of the oligonucleotide solution to obtain target-response curves. Solutions were incubated at room temperature for ∼40 min in the dark. All solutions were prepared in PBS buffer with additional 2 mM MgCl_2_ and samples were analyzed in triplicate in 384-well black plates using a Victor II microplate reader (PerkinElmer, Waltham, MA) with FAM excitation/emission at 480 nm/525 nm. The oligonucleotides were purified by reversed-phase HPLC and were obtained from Integrated DNA Technologies (Coralville, IA, U.S.A.).

### OWLS Measurements

The OWLS 210 instrument (MicroVacuum Ltd., Hungary) was used to investigate DNA-peptide interactions in a sequence and surface-specific manner. Measurements were conducted in a flow cell to extract the surface binding kinetics and affinities. Optical waveguide sensor chips (OW2400, MicroVacuum Ltd., Hungary) were plasma cleaned (2 min at 200 W) and prefunctionalized with aptamers using the functionalization method mentioned above. Upon insertion of the chip into the instrument, 1× PBS buffer was introduced into the flow cell. Mode spectra yielding the effective refractive indices of the zeroth transverse electric (*N*_TE_) and transverse magnetic (*N*_TM_) modes were measured until stable values in the running buffer were achieved. Then, different peptide solutions in the same buffer (1× PBS) were flown into the system, and peptide–aptamer interactions were monitored in real time. Upon observing saturated binding, the peptide solution was replaced with fresh 1× PBS to test for peptide desorption from the DNA monolayers. The adsorbed surface mass density values were determined using the de Feijter’s formula^60^ with a d*n*/d*c* = 0.182 g/cm^3^.^61^

### Peptide Sensing

A 0.29 mM solution of porcine Dynorphin A (≥95% (HPLC), ≥65 wt %, 2.15 kDa, Sigma-Aldrich) was stored in 10 μL aliquots at −20 °C and thawed prior to use. The Control and Phe peptides were synthesized by LifeTein (Randolph, NJ, U.S.A.) and shipped in lyophilized form. From each, aliquots were prepared by dissolving the protein in MQ water. The aliquots were stored at −80 °C. Further information on the peptides and stock concentration of the aliquots are shown in Table 2.

**Table 2:** Peptide Sequences, Properties, and Technical Values Given by the Suppliers and the Net Charge Taken from pepcalc.org

peptide name	amino acid sequence	molecular mass (kDa)	stock concentration (mg/mL)	net charge at pH 7	purity (%)
porcine dynorphin A (Dyn)	Tyr-Gly-Gly-Phe-Leu-Arg-Arg-Ile-Arg-Pro-Lys-Leu-Lys-Trp-Asp-Asn-Gln	2.15	0.2	+4e	≥95
negative control (control)	Ser-Gly-Thr-Trp-Trp-Tyr-Tyr-Ile-Asn-Thr-Gly-Gly-Arg-Arg-Arg-Arg-Arg	2.19	0.5	+5e	98.16
single stretch (Phe)	Ser-Gly-Thr-Phe-Phe-Phe-Phe-Ile-Asn-Thr-Gly-Gly-Arg-Arg-Arg-Arg-Arg	2.08	0.5	+5e	98.14

A peptide suction technique was established to get the peptides into the cantilever. The back reservoir of the cantilever was filled with 10 μL of pure PBS and the pneumatic connector was attached and sealed with wax afterward. The cantilever was then mounted on a Teflon holder that had a reservoir (pretreated with BSA to avoid nonspecific adsorption of the peptides) and filled with 100 μL of stock solution. By application of −800 mbar for a time span of around one h, the peptide was sucked into the pore and the cantilever. After suction, the pore was mounted onto the AFM scan head, and measurements were conducted. With this technique, it took between 5 and 30 min for the peptides to reach the pore and generate signals. This method was used for all of the peptides.

### Data Analysis

An explanation of the continuous wavelet transformation-based data analysis is presented in the Supporting Information.

## Supporting Information Available

Interface nanopore formation model (Figure S1); chemical structures and properties of peptides (Table S1); 3-D structure of peptides (Table S2); contact angle to validate surface chemistry (Figure S2); control measurements of BSA passivation (Figure S3); statistics for OWLS measurements and current–voltage curves of silanized vs. aptamer-modified substrates (Figure S4); schematic of OWLS mechanism (Figure S5); current traces of Dyn translocations on aptamer-modified substrates (Figure S6); wavelet transformation and spike detection (Figure S7); classification of identified signal changes (Figure S8); current traces for translocation of Control peptide (Figure S9); translocation characterization of Control peptide (Figure S10); clustering and peak analysis of Control peptide (Figure S11); clustering and peak analysis of Phe peptide translocations through aptamer-functionalized surfaces at 1.5 V (Figure S12); clustering and peak analysis of Phe peptide translocations through scrambled DNA-functionalized surfaces at 1.5 V (Figure S13); geometry of COMSOL simulations (Figure S14); electric field and ion concentrations inside the nanopore (Figure S15); COMSOL simulations of peptide translocation through 2 nm pore (Figure S16); current level extraction of a computed current signal (Figure S17); parameters for single amino acids in signal generation (Table S3); motif search by autocorrelation of a sliding window and virtual signal from peptide sequence (Figure S18) (PDF)
