# Supplementary material for: Aptamer-Functionalized Interface Nanopores Enable Amino Acid-Specific Peptide Detection
Source: ACS Nano. 2024 Feb 14;18(8):6286–97. doi: 10.1021/acsnano.3c10679 (PMC10906075; doi:10.1021/acsnano.3c10679)
Supplement: Supplementary file 1 — nn3c10679_si_001.pdf [file nn3c10679_si_001.pdf]

Supporting Information

Aptamer-Functionalized Interface Nanopores Enable Amino Acid-Specific Peptide Detection

Tilman Schlotter,[1] Tom Kloter,[1] Julian Hengsteler,[1] Kyungae Yang,[2] Lijian Zhan,[1] Sujeni Ragavan,[1] Haiying Hu,[1] Xinyu Zhang,[1] Jens Duru,[1] János Vörös,[1] Tomaso Zambelli,[1] and Nako Nakatsuka[1*]

[1] Laboratory of Biosensors and Bioelectronics, Institute for Biomedical Engineering, ETH Zürich, 8092 Zürich, Switzerland

[2] Department of Medicine, Columbia University Irving Medical Center, New York, NY 10032, USA.

* Corresponding author

S1. Interface Nanopore Formation Model

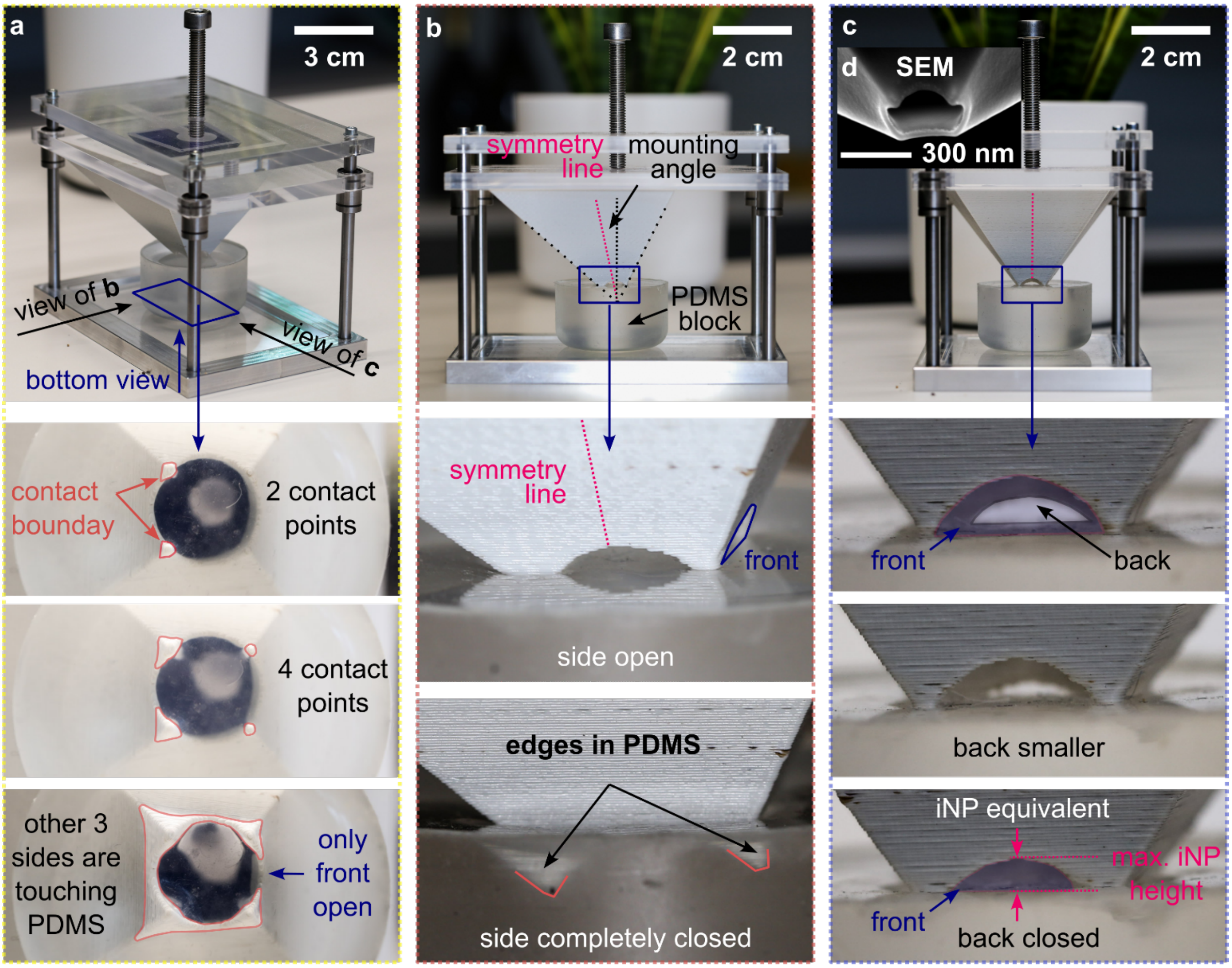

Figure S1: Equivalent model of the interface nanopore formation in a 1:50,000 ratio. a, image on the mode where the applied pressure influences the size of the nanopore at the interface of a soft substrate. The images from the bottom show three different positions of the apex tip sinking into the PDMS: from first contact (top), to interfacing all four tips of the cantilever (middle), to connecting with PDMS of all apex edges besides the front opening to form a single nanopore**. b,** side view of model perpendicular to tilting angle; below closure of side openings with increasing pressure of the tip against the PDMS. **c,** view from the front showing closure of the back end and reduction of iNP size with increased applied pressure. **d,** scanning electron microscopy image of the silicon-fabricated cantilever tip used in model.

S1.1. Interface nanopore model fabrication

A macroscale force-controlled interface nanopore (iNP) setup was formed at the interface between a soft polydimethylsiloxane (PDMS) substrate and the aperture of a Si_3N_4 micro-channeled AFM cantilever, creating a nanoconfined area. For clear visualization of the geometry changes of the iNP at different forces, we fabricated a 50,000x (side to side 1.5 cm, corner to corner 1.1 cm) scaled-up model (**Figure S1a-c**). The original probe has a 10 × 10 × 7 μm^3 pyramid at the extremity with an aperture of 300 nm diameter at its apex. The aperture edge is not a planar surface, but it is characterized by four sharp corners that form arches with a maximum height of 20−50 nm along the perimeter (**Figure S1d**, SEM image). The 3D printed tip had the same shape as the one used in our experiments. The pyramid has been rotated by 11° (**Figure S1b**, mounting angle of 11° from vertical black line while in **Figure S1c,** the red symmetry line corresponds to the black vertical one), similarly to the cantilever that was mounted on an AFM head also with an angle of 11° in our set-up. When the tip was vertically moved down and contacted the PDMS substrate with an applied force, a macroscopic version of the iNP forms. While increasing the applied force, we could see that the arch on the front face of the pyramid-shaped tip was first closed, then the two side faces subsequently closed, leaving just the back face still open for translocation of biomolecules.

S2. Supplementary Tables

Peptide name	Amino acid sequence (1 and 3 letter)	Molecular mass [kDa]	Stock concentration [mg/ml]	Net Charge at pH 7	Purity
Porcine Dynorphin A (Dyn)	NH2- YGGFLRRIRP KLKWDNQ -COOH	2.15	0.2	+4e	≥ 95 %
	Tyr-Gly-Gly-Phe-Leu-Arg-Arg-Ile-Arg-Pro-Lys-Leu-Lys-Trp-Asp-Asn-Gln				
	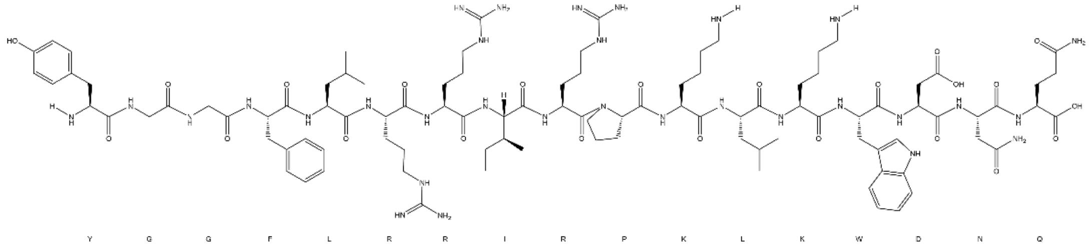				
Negative control (Control)	NH2- SGTWWYYINT GGRRRRR -COOH	2.19	0.5	+5e	98.16 %
	Ser-Gly-Thr-Trp-Trp-Tyr-Tyr-Ile-Asn-Thr-Gly-Gly-Arg-Arg-Arg-Arg-Arg				
	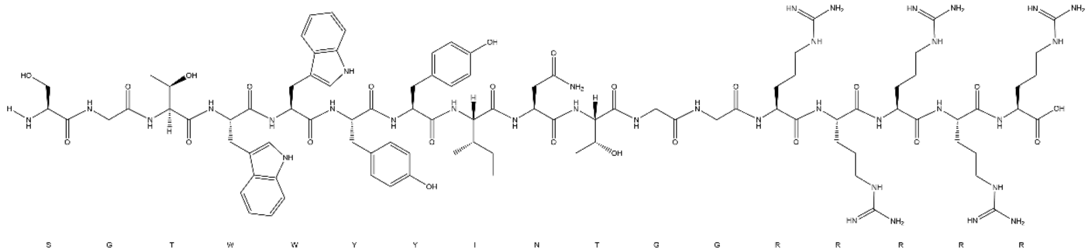				
Single stretch (Phe)	NH2- SGTFFFFINT GGRRRRR -COOH	2.08	0.5	+5e	98.14 %
	Ser-Gly-Thr-Phe-Phe-Phe-Phe-Ile-Asn-Thr-Gly-Gly-Arg-Arg-Arg-Arg-Arg				
	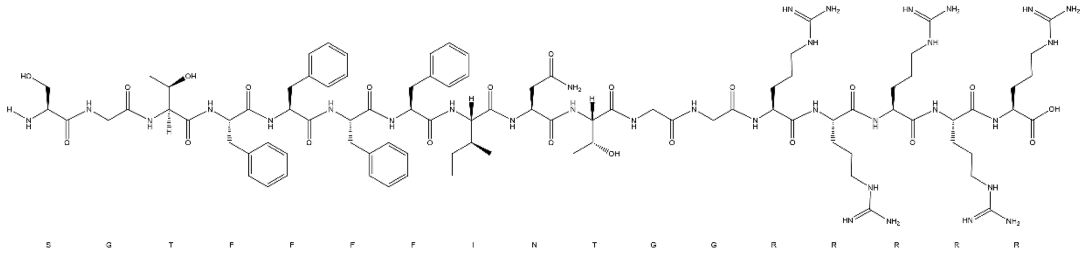				

Table S1: Chemical structures and properties of the peptides used in this manuscript. The chemical structures were made in ChemDraw.

Porcine Dynorphin A (Dyn)

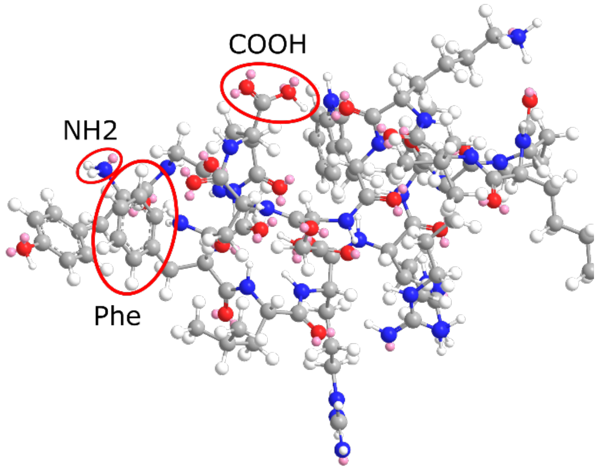

Negative control (CTRL)

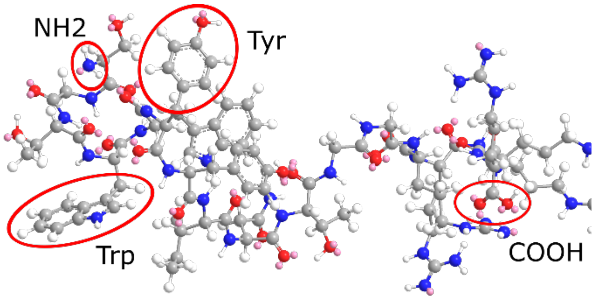

Single stretch (Phe1)

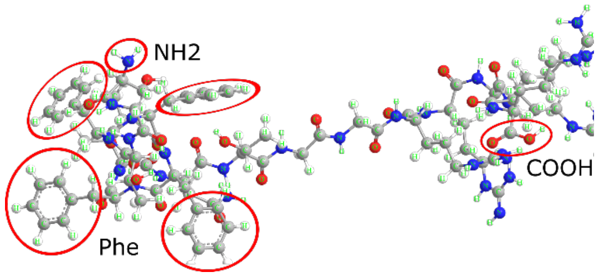

Table S2: 3D-structure of peptides used in this manuscript. The 3D structures were generated with Chem3D (PerkinElmer, USA) using the MM2 energy minimization algorithm provided in Chem3D.

S3. Supplementary Methods

S3.1. Verification of covalent DNA immobilization

Since each step of the functionalization procedure introduces polar functional groups on the surface, hydrophilicity of the substrate increases throughout the process. The increase in hydrophilicity upon each step was monitored by observing the water contact angle. On untreated PDMS, a 100 µl MQ water droplet had a 90° contact angle as depicted in Figure S2. Upon silanization and MBS treatment, water contact angles got smaller and reached a minimum of around 20° after DNA immobilization (**Figure S2b, c,** and **d**, respectively). This change in hydrophilicity was also observed from the top view (**Figure S2e - h**).

Further confirmation of DNA immobilization was gathered by DNA staining with the fluorescent dye SYBR gold (Fig. 1). Under a light microscope, functionalized slides showed a cracked surface. Those surface cracks are a result of n-hexane treatment, which removed excess uncured residues in the PDMS-coated glass slides. When submersed in n-hexane, the PDMS layer expanded and subsequently contracted again when the n-hexane was removed, leading to mechanical tearing of the substrate. The tearing force led to crack formation, but the areas in between presented a smooth surface for nanopore approaches. This surface structure was also observed in fluorescent images as seen in Fig. 1c and d.

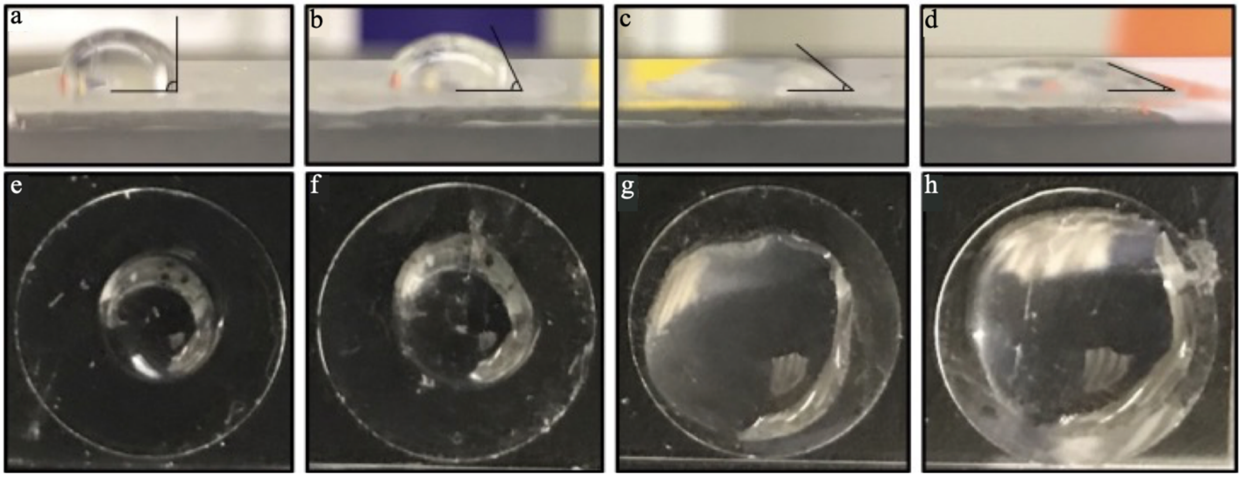

Figure S2: Water contact angle measurement for functionalization steps. a-d, Side view of a 100 µl MQ water droplet on PDMS surfaces that are **a,** untreated, **b,** silanized, **c,** MBS-functionalized, and **d,** aptamer-functionalized. Water contact angles are indicated with angle signs. Below: Top view onto the corresponding upper slide.

S3.2. Surface passivation of Teflon reservoir does not yield nonspecific translocations

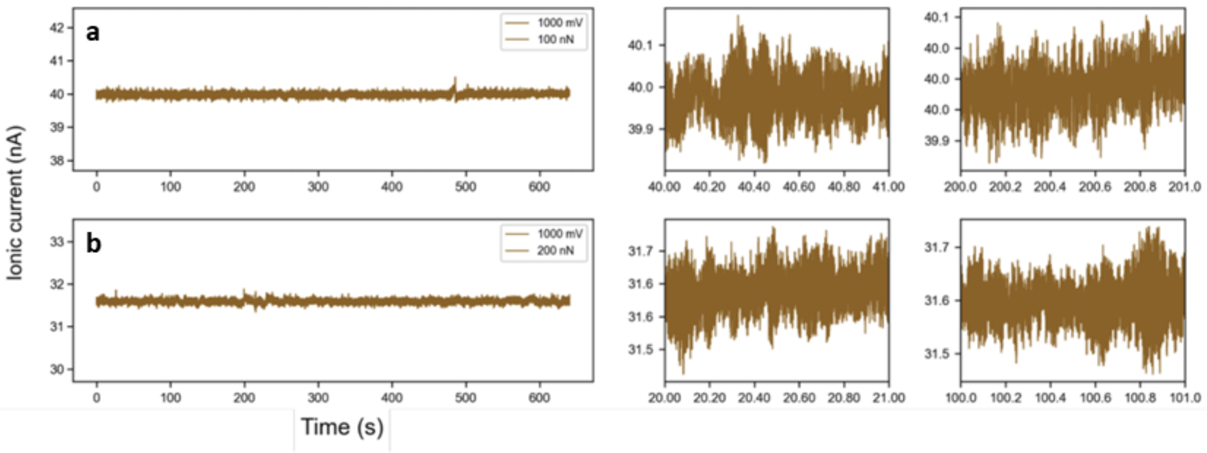

Figure S3: Control measurements to ensure no free bovine serum albumin (BSA) is present in the reservoir post-passivation. Data in **a** and **b** were collected within the same measurement series on different locations on aptamer-modified PDMS at 1.0 V. Instead of the peptide solution, only PBS was filled into the Teflon holder reservoir. Aspiration and measurements were conducted in the same manner as typical experiments in the main text. This control test confirmed that negligible free BSA remained in the reservoir post rinsing, and that the passivation treatment of the Teflon reservoir does not generate translocation events.

S4. Supplementary Figures

S4.1. Specific and non-specific binding of peptides to aptamers

For statistical analyses, **Figure S4** shows the end points (signal saturation) of the transient optical waveguide lightmode spectroscopy (OWLS) curves of **Figure 2b-d**. The Phe peptide on the Phe aptamer-functionalized OWLS chips show significant increase in bound mass compared to the controls and Dyn peptide. No significant difference was observed between the two control chips: the control peptide on Phe aptamer-modified chips and the Phe peptide tested on scrambled DNA chips. The Dyn peptide binding on the OWLS chip is significantly lower, which can be attributed to the combination of less Phe moieties (one in Dyn *vs.* four in Phe1) and a lower net charge (+4*e* for Dyn *vs.* +5*e* for the Phe1 and the Control peptide).

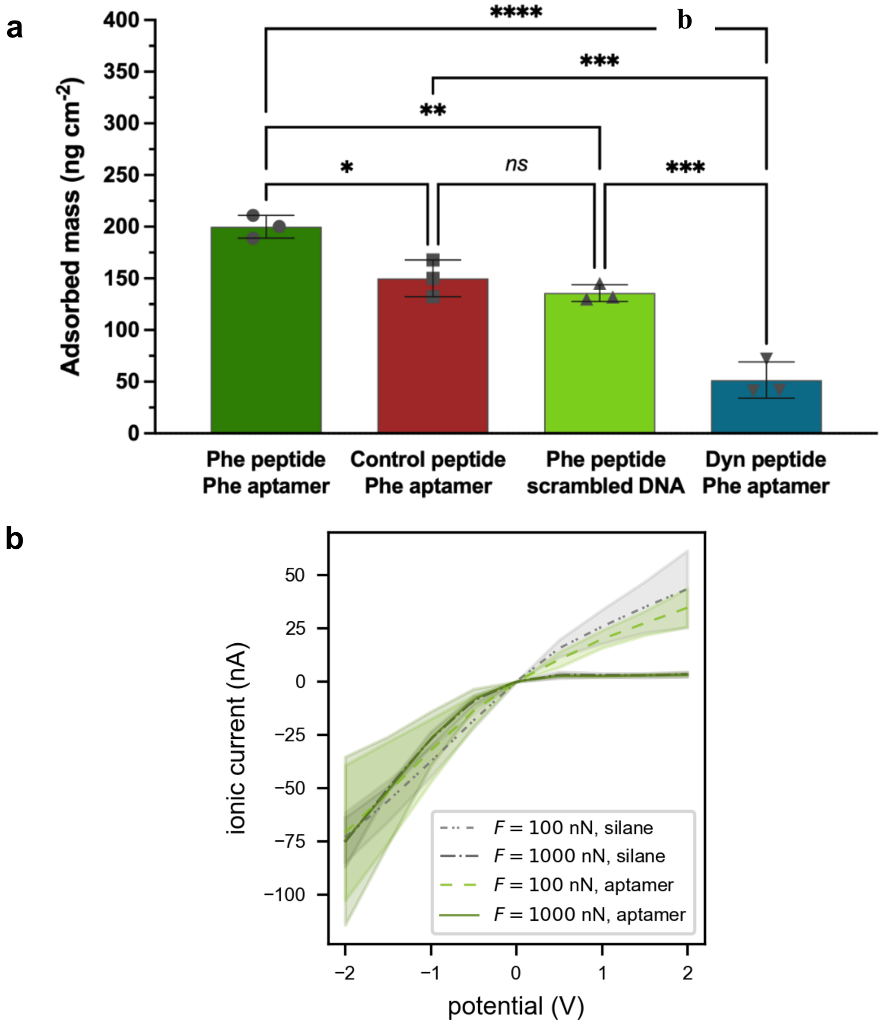

Figure S4: Optical waveguide lightmode spectroscopy (OWLS) surface binding. a, Mass bound to OWLS chip for three different peptides (Phe, Control, Dyn) on either aptamer-modified or scrambled DNA-

functionalized OWLS chips. The mean mass absorption and the standard deviation are shown for three different chips (N=3). A one-way ANOVA was performed to compare the interactions between the different groups tested. Statistically significant differences were observed in the mean adsorbed mass values for at least three groups ($F(3,8) = [56.03]$, $p<0.0001$). Tukey's test for multiple comparisons found that the mean was significantly different between Phe peptide+Phe aptamer *vs*. all other groups with varying p values indicated in the plot where *: $p<0.05$, **$p<0.01$, ***$p<0.001$, and ****$p<0.0001$. There was no statistical significance in the mean between the two controls tested (*ns*: not significant, p=0.63). **b,** Current-voltage curves of silanized vs. aptamer-functionalized substrates, measured at forces of 0.1 and 1.0 µN including the standard deviation range (N=3).

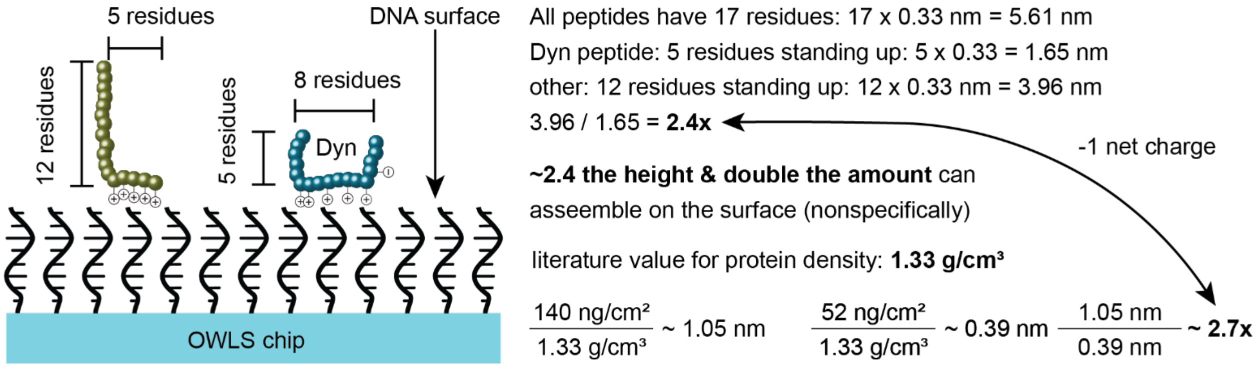

Figure S5: Explanation of difference in binding between Dyn and other peptides. Figure shows a schematic of peptides being bound to DNA surface. Right Dyn containing 4 net charges, left the control and Phe peptide with 5 positive net charges being bound nonspecifically to the DNA surface.

Figure S6 shows long ionic current traces of translocations on Phe aptamer functionalized substrates as depicted in **Figure 2e**. Each of the events (A, B, C, D) represents binding to, movement inside, and unbinding of the peptide within the nanopore.

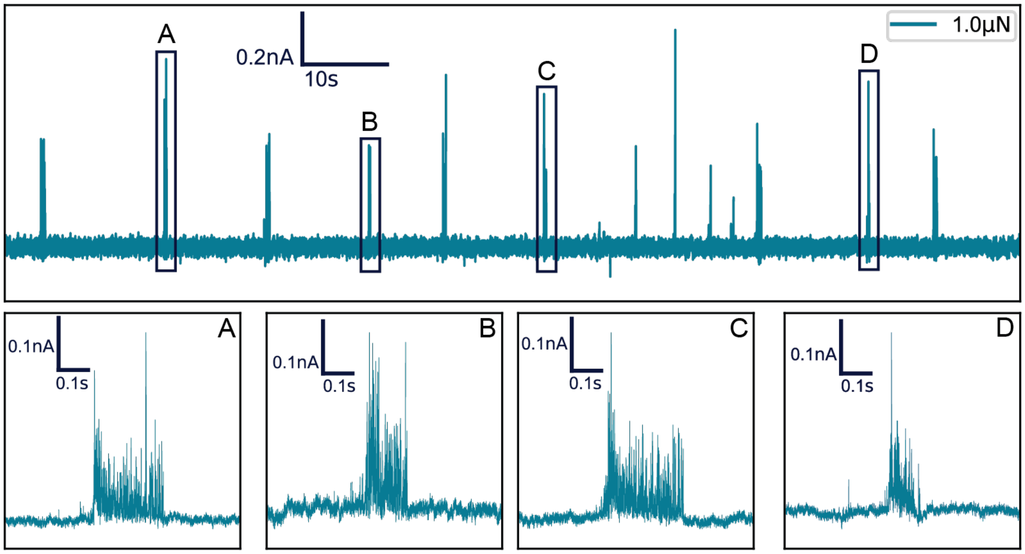

Figure S6: Current traces of Dyn translocations on Phe-aptamer functionalized substrate. Current traces were recorded at a bias potential of 1V and an applied force of 1.0 μN with a sampling rate of 40 kHz. Subfigures A, B, C, and D show magnifications of the current trace with the corresponding window in the upper subfigure.

S4.2. Comparison of effect of number of Phe moieties on current traces between Dyn and Phe-peptide

As Dynorphin translocations were presented in Figure 4a-e of our previous work,[1] we used Dyn again to benchmark the functionalized iNP against previous results as shown in **Figure 2h**. From there on we tested the conditions for optimal specific interaction of the translocating peptides with the immobilized aptamers. Retention times showed that the interaction is higher at higher forces **Figure 2h-j**. For further measurements a peptide with four Phe moieties and a control peptide with Phe moieties being replaced by Trp and Tyr were chosen.

We saw that the presence of four compared to just one Phe-moiety in the backbone had a significant impact on both the translocation time as well as the ionic current trace generated upon translocation. As shown in current traces of **Figure S6** and density plots of **Figure 2j**, translocations of Dyn peptides are in the range of 70 ± 124 ms (mean and standard deviation) and only current enhancement is observed. Translocations of Phe-containing. **Figure 4e, h,** and **k** show translocation times above 1 s and both a current enhancement followed by a current drop (also see **SI section S4.6**) is observed. In addition, **Figure 4** shows distinct current levels that were not observed for Dyn translocations supporting the hypothesis that they originate from peptides hopping from one aptamer to another one.

S4.3. Continuous wavelet transformation for change point and event detection

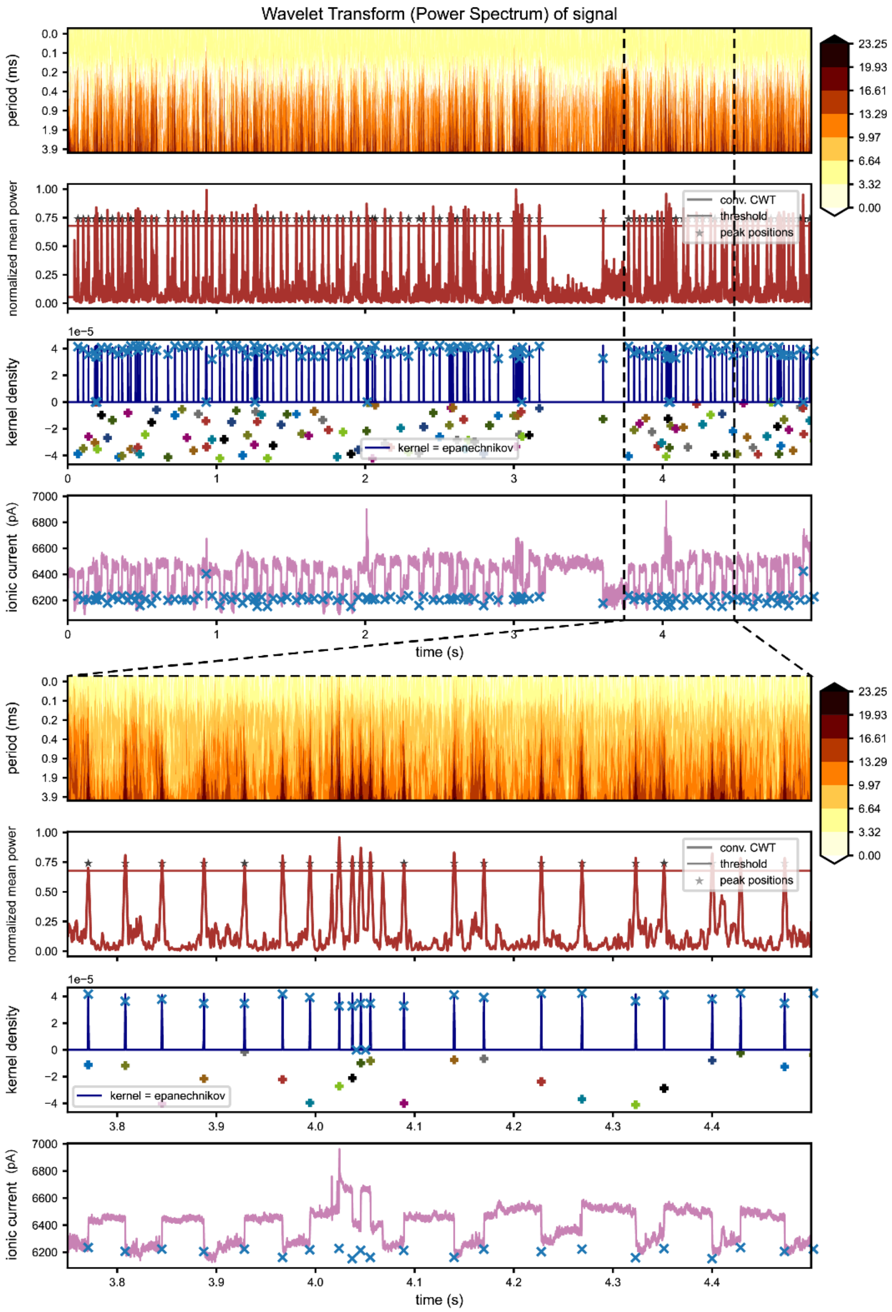

Figure S7: Wavelet transformation and spike detection. The current trace is shown in the lowest figure (purple line) and the corresponding wavelet transformation is shown as a contour plot in the top figure. Below, (red line) the normalized signal of the mean continuous wavelet transformation (CWT) is shown with the identified peak positions marked by a star. The kernel density estimation (blue line) of the peak positions (colored crosses corresponding to the categories defined in the text and figure below) distribution and the resulting cluster peak positions (blue cross) are shown . Bottom figures are zoom-ins of top figures.

Classical thresholding approaches for event detection cannot be applied to the data as it causes too many errors. Therefore, spike detection *via* continuous wavelet transformation[2,3] is applied to detect the rapid changes in the signal (**Figure S7**). Each detected peak is subsequently assigned to one of four categories:

0 The kernel density estimation cluster has only one peak identified and a total gradient below 0.25 and above -0.25 and is therefore seen as a spike.

-1 The kernel density estimation cluster has only one peak identified and a total gradient below 0.25 and is therefore classified as a drop.

1 The kernel density estimation cluster has only one peak identified and a total gradient below 0.25 and is therefore classified as an increase.

2 The kernel density estimation cluster identified more than one peak.

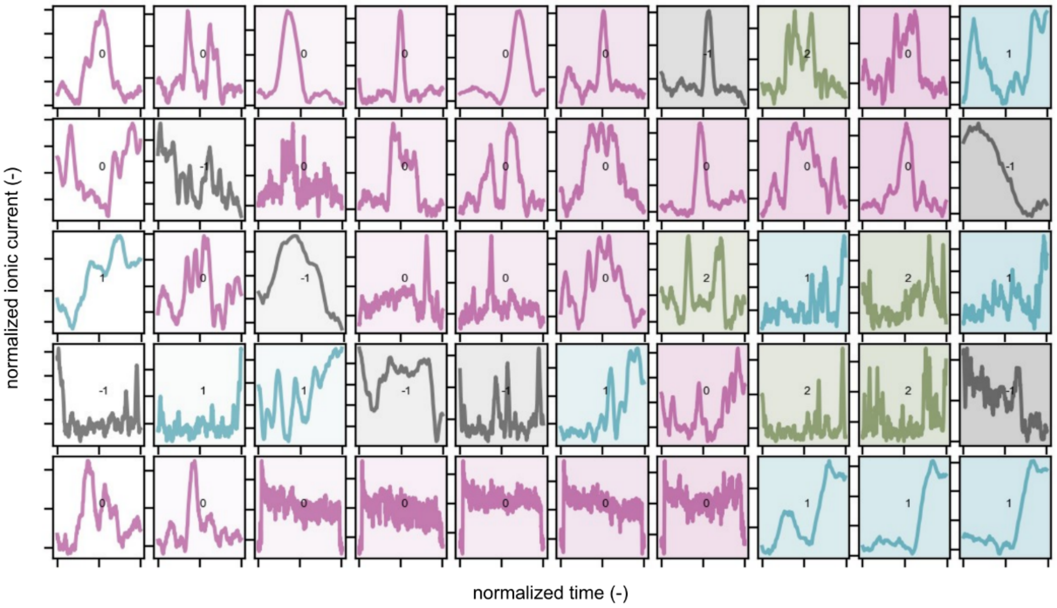

Figure S8: Classification of identified signal changes. Blue panels show an increase in current (1), black panels show a decrease in current (-1), purple panels show an event with both increases and decreases (0). Green panels could not be assigned to any of the prior categories (2).

Afterwards, an algorithm identifies the individual translocations based on the information extracted. The software is written in Python 3.

S4.4. Time series recording of control peptide and translocation characterization

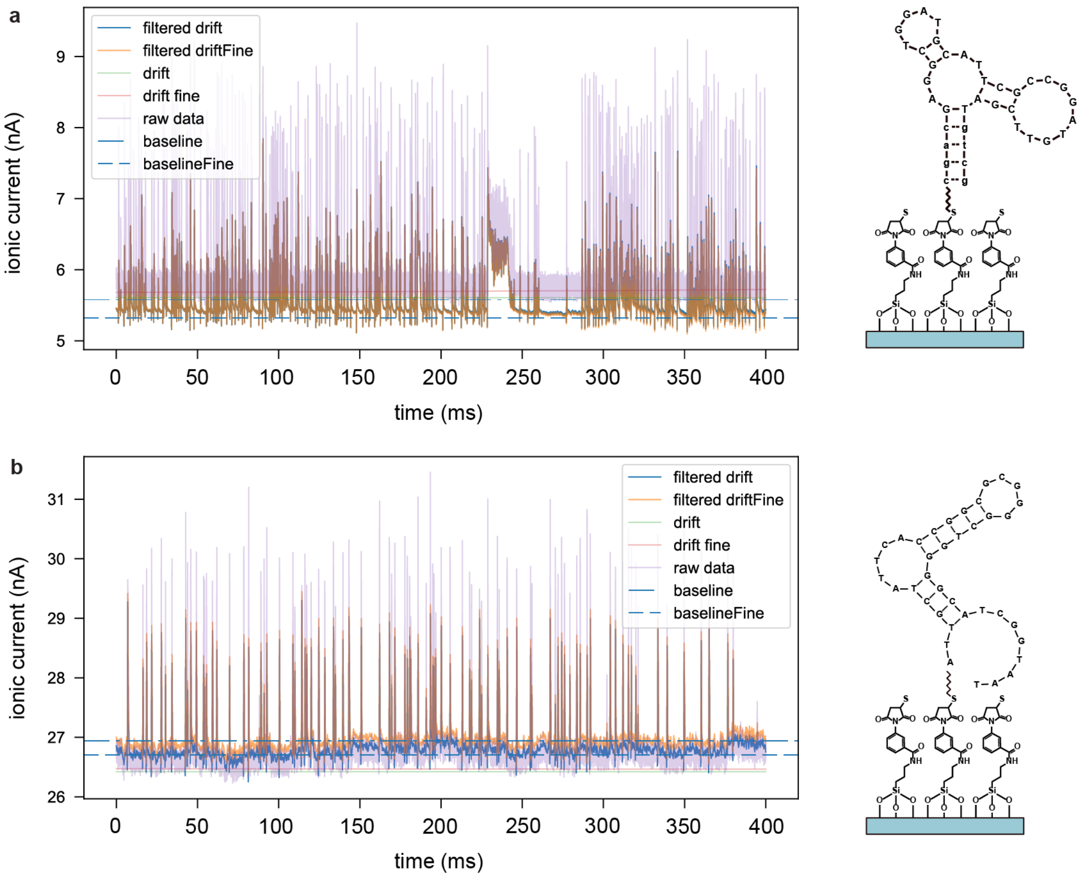

Figure S9: Current traces of translocation measurements of the Control peptide. a, signal obtained from peptide translocations on substrates with the specific aptamer functionalization. **b,** signal obtained on substrates modified with the scrambled sequence. The figures show the data after several filtering steps. The purple line represents the unfiltered raw data. A long-term and a fine drift in the data is extracted using an ALS[4] algorithm and the median of the drift signal is used as the corresponding baseline current. A filtered signal is created using a Savitzky-Golay filter with a window of 20 data points. Subsequently, the drift (or drift fine) is subtracted from the signal and the baseline (or baselineFine) is added again.

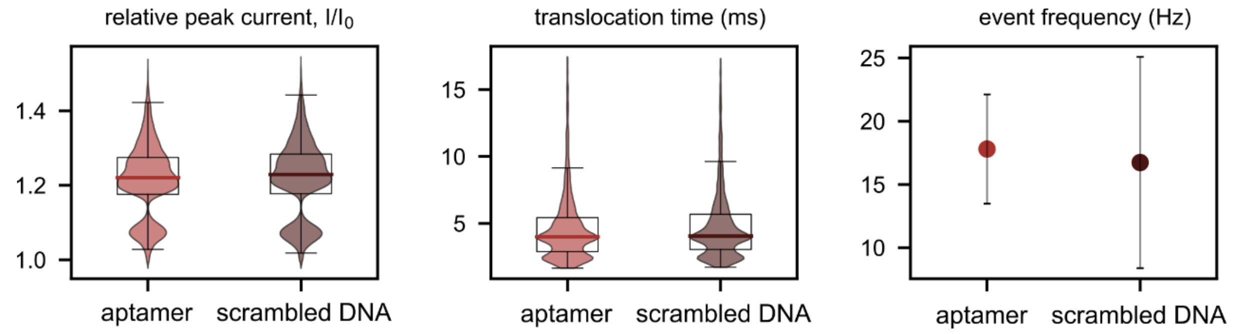

Figure S10: Translocation characterization of the Control peptide. Violin plots of peak currents, translocation times (as shown in the density plot of **Fig. 3c**), and translocation frequencies (including the standard deviation) for the Control peptide through both Phe aptamer- and scrambled DNA-functionalized interface nanopores. The translocations of Control peptides on the two different substrates do not show significant differences in translocation characteristics.

S4.5. Shape analysis of peptide translocations

Dimension reduction either by a principal component analysis (PCA), t-distributed stochastic neighbor embedding (t-SNE)[5,6], or uniform manifold approximation and projection for dimension reduction (UMAP)[7] were used to identify patterns in translocation events. A subsequent clustering of the dimension reduction analysis was done both by density-based cluster scanning (DBSCAN)[8] and by ordering points to identify the clustering structure (OPTICS)[9], which are two closely related clustering algorithms.

DBSCAN, OPTICS, PCA, and t-SNE are part of the *sklearn* package of *Python3* and for the UMAP algorithm an own package is available (https://umap-learn.readthedocs.io/en/latest/).

The shape analysis was always a combination of a dimension reduction analysis (either PCA, t-SNE, or UMAP) followed by a clustering (DBSCAN or OPTICS), which yields six different analysis possibilities as shown in **Figure S11**. The PCA analysis (for the first two principal components) did not yield clusters compared to t-SNE or UMAP. Between the latter two, UMAP yielded better results within a shorter computation time. OPTICS yielded a finer clustering compared to DBSCAN which is why the preferred combination was a UMAP dimension reduction followed by an OPTICS clustering.

The identified clusters are shown in subsequent plots where the x- and y-axes represent the two dimensions in the UMAP and t-SNE space. If an event was not attributed to a cluster, it was considered as "noise". The events not attributed to a cluster were classified as "-1" (**Figure S12**, **Figure S13**). As the events were

stretched to equal length prior to analysis, mean shape calculation could be done for each cluster by averaging all cluster-specific events. Each mean shape of a cluster was plotted in a different color (**Figure S11**). The shape analysis showed different peak characteristics for Phe peptide translocations on aptamer-functionalized substrates and all the other translocation configurations.

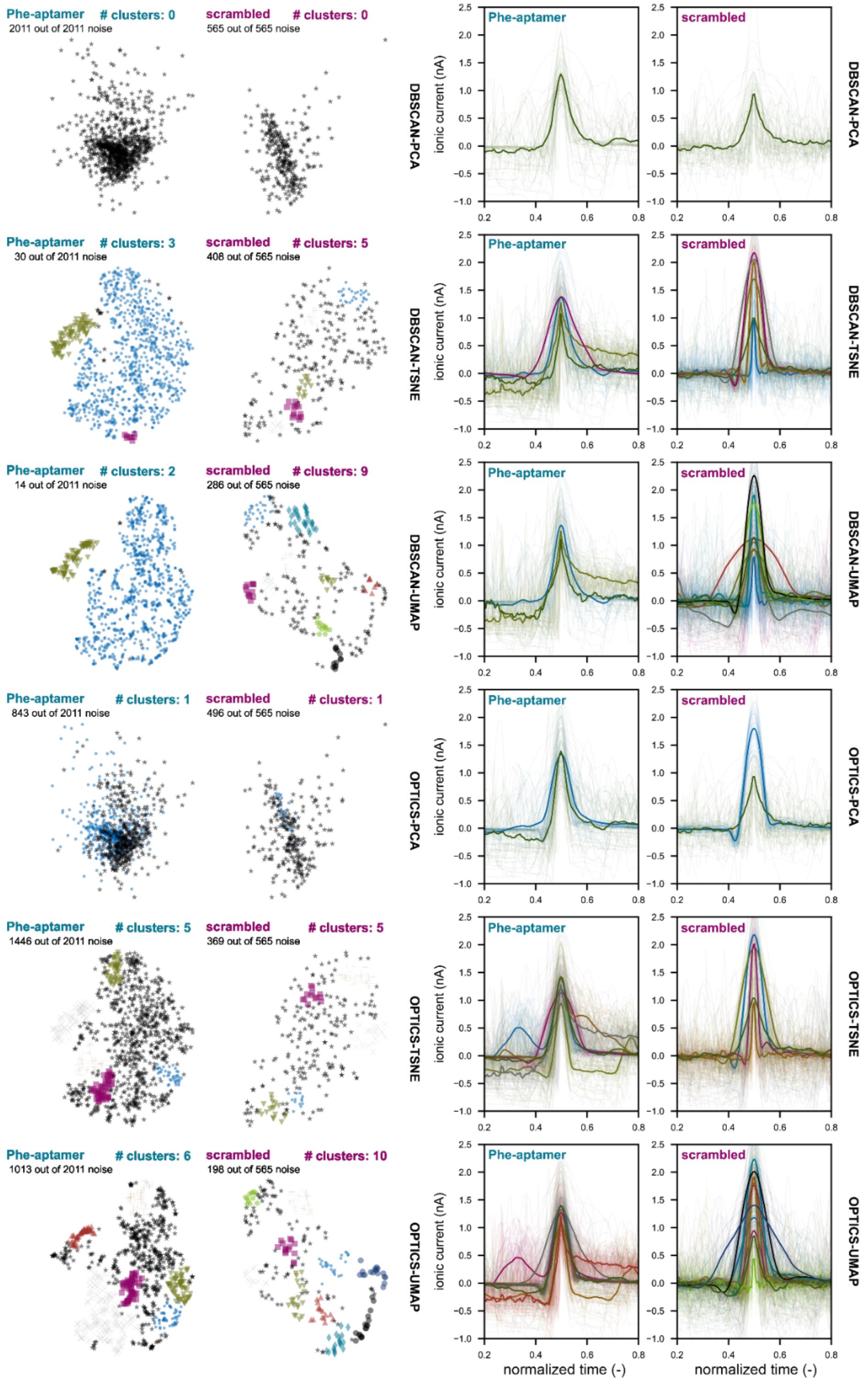

Figure S11: Clustering and peak analysis of the Control peptide. On the left side, the results of the different clustering algorithms are shown for both the translocation through a scrambled and an aptamer-modified interface nanopore. The noise gives the amount of translocation events that could not be categorized into one cluster (black dots). Principal component analysis (PCA) with DBSCAN yields only one cluster (all events are categorized as “noise” that are the “-1” cluster that can be seen on the right side).

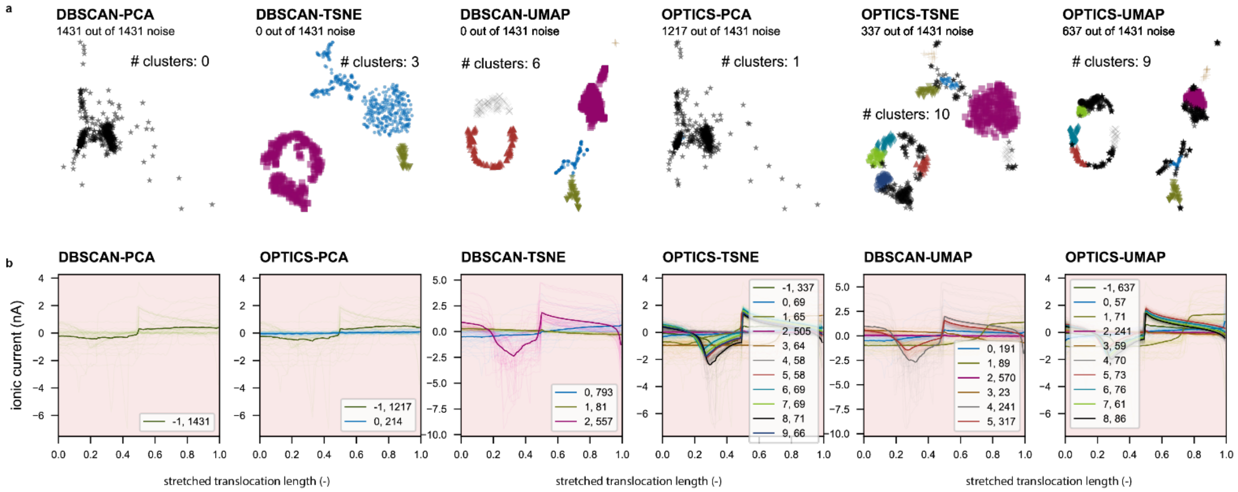

Figure S12: Clustering and peak analysis of Phe peptide translocations through aptamer-functionalized surfaces at 1.5V. a, Color-coded clusters analyzed with OPTICS or DBSCAN clustering algorithms. **b,** Corresponding ionic currents and cluster-mean-shapes. The legend in **b** shows the cluster number and the number of events that are attributed to that specific cluster.

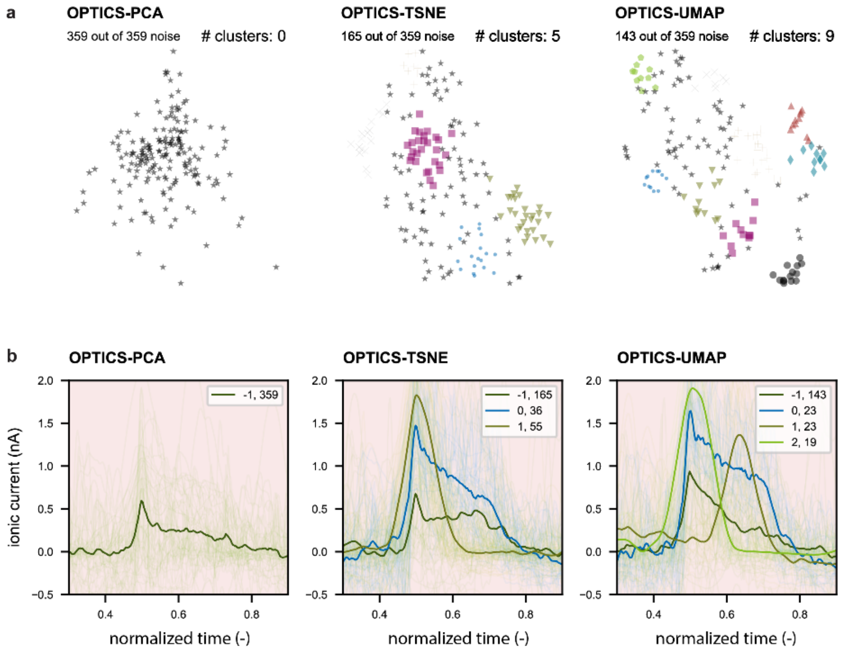

Figure S13: Clustering and peak analysis of Phe peptide translocations through scrambled DNA-functionalized surface at 1.5V. a, Color-coded clusters analyzed with OPTICS clustering algorithms. **b,** Corresponding ionic currents and cluster-mean-shapes. The legend in **b** shows the cluster number and the number of events that are attributed to that specific cluster.

S4.6. COMSOL simulations

A COMSOL simulation was performed based on the model of Zeng *et al.*[10] As the iNP is created by interfacing a soft substrate with the FluidFM, the simulation was adapted for a non-rotational symmetric 2D simulation. A 2D simulation was chosen due to the complexity of the simulation resulting in computational limitations. Similar to our prior publication,[1] the geometry was created by subtraction of a circle with a radius of 340 nm from a hollow 11° tilted triangle with a thickness of 80 nm that represents the cantilever wall thickness (see **Figure S14a**). Afterwards, a simulation domain is defined (**Figure S14b**), which includes one side of the FluidFM cantilever wall (**Figure S14c**) and in the nanogap region, a 17-residue peptide is emulated by subtracting 17 circles next to each other each with radii of 0.5 nm (**Figure S14d**). The height of the gap between the cantilever was set to 2 nm. A fillet was added to the corner of the cantilever wall with a radius of 0.5 nm.

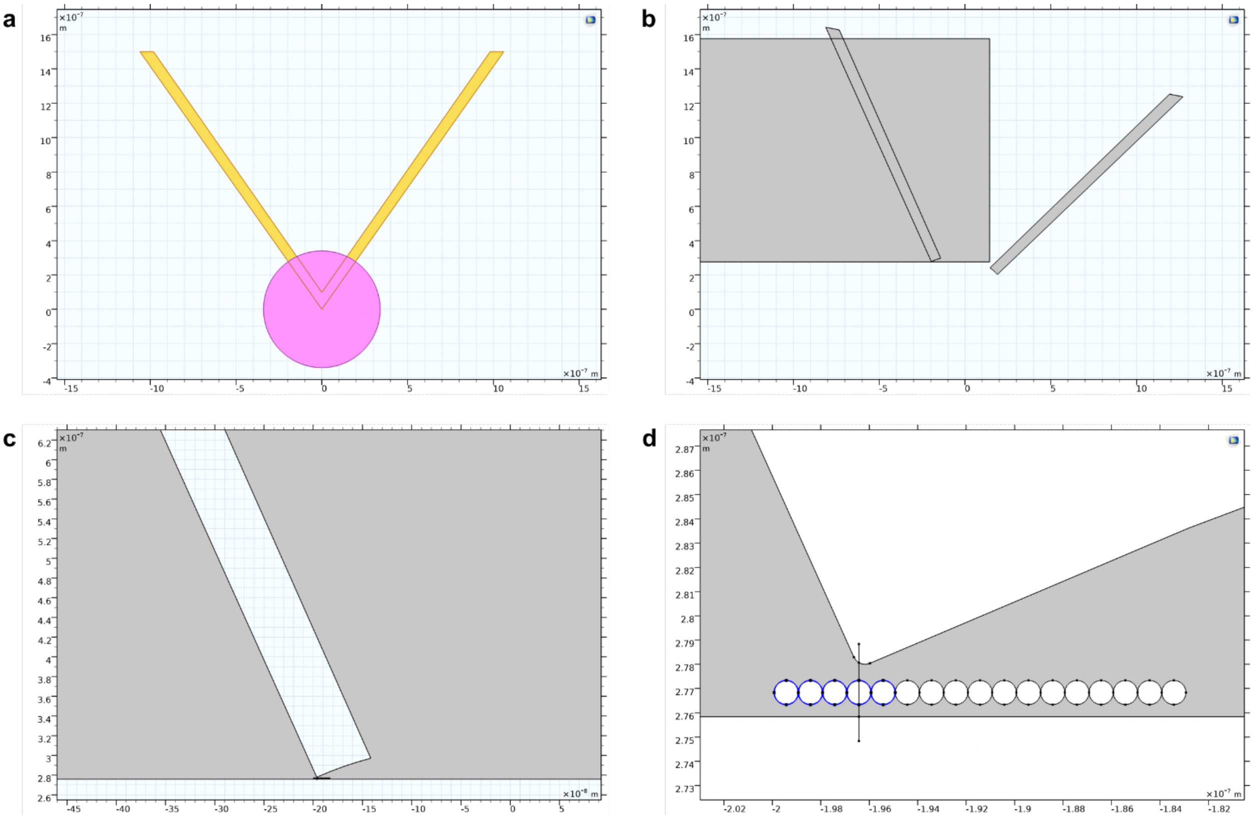

Figure S14: Geometry of COMSOL simulations. a, Shows the hollow triangle with a subtracted circle. **b,** Shows the simulation domain. **c,** one side of the cantilever wall. **d**, Magnification of nanogap with a chain of 17 circles of 1 nm diameter. The gap was set to 2 nm.

A Multiphysics model is created including the modules Transport of Diluted Species, Electrostatics, and Laminar Flow. Two species are added representing Cl^- and K^+ ions, respectively. Diffusion coefficients, D, were user defined with values 1.8e-9 m^2/s and 1.87 m^2/s and the mobility of both species is calculated using $\mu = \frac{D}{R\,T}$. The solution concentration at the inlet and outlet is set to 150 mol/m^3. In the electrostatics module, the top boundary inside of the cantilever was set to a potential of 1 V and the left edge of the simulation domain was set to ground. Surface charges on the cantilever wall were set to 0.01 C/m^2, on the amino acid circle 0.02 C/m^2, and on the bottom substrate 0.03 C/m^2. The space charge density was user defined as $q * N * (c_1 * z_1 + c_2 * z_2)$ with $c_{1/2}$ being the concentrations of species 1 (K^+) and species 2 (Cl^-) and $z_{1/2}$ the corresponding ion charge, and *N* the Avogadro constant. In the laminar flow module, an inlet on the left side, an outlet inside of the cantilever, and a volume force in x $(c_1 - c_2) * N * q * es.Ex)$ and y-direction $(c_1 - c_2) * N * q * es.Ey)$ was defined. The *es.Ex/Ey* are vector components of the electrical field in the x- and y-direction, respectively. The mesh is physics controlled and the element size was set to "extra fine". The solution is a stationary solver with a parametric sweep of the peptide position. The ion concentrations and the electric field inside the nanopore without peptides present inside the pore is shown in **Figure S15**.

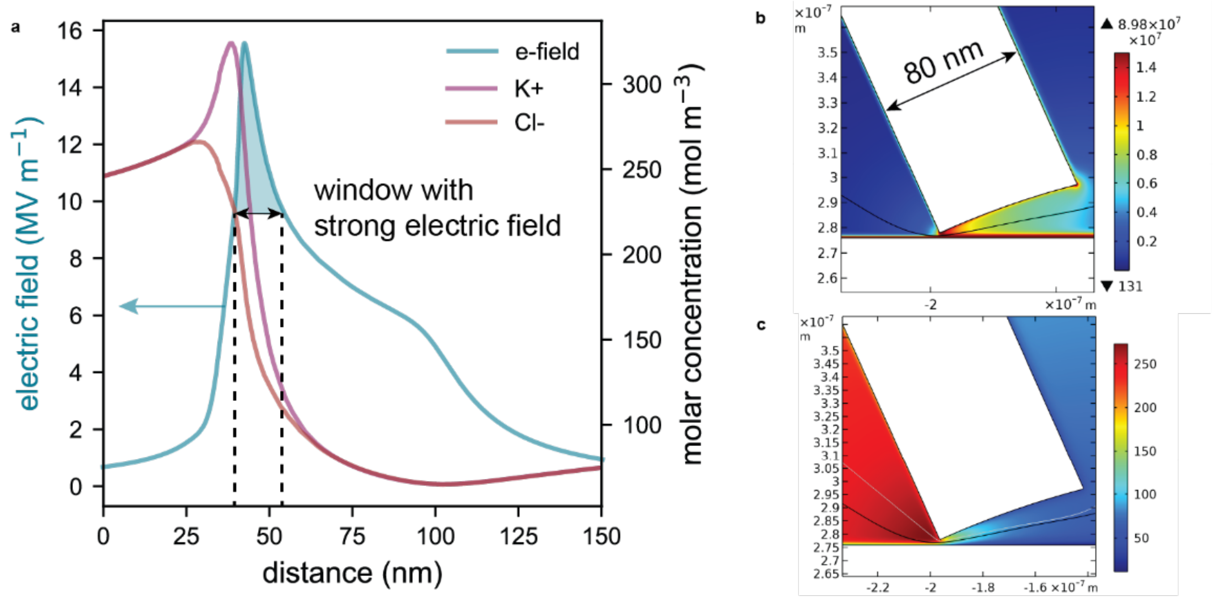

Figure S15: Electric field and ion concentrations inside the nanopore. A, Plot of the electric field (blue line, left y-axis) and the corresponding ion concentrations in a 150 mM KCl solution. Red and purple lines correspond to the Cl^- and K^+ concentrations. **B**, 2D surface plot of the electric field. **C,** 2D surface plot of the Cl^- concentration inside the nanopore. The line plot shows the data along the black line in **b** and **c**.

To simulate the difference between a translocation where the positive charges enter the iNP first (forward, from the C-terminus side) *vs.* where the charges enter the iNP last (backward, from the N-terminus side), the current through the iNP with respect to the amino acid-position of the middle amino acid (which is the 9th moiety in the backbone) was calculated. Since the peptide is positively charged, it translocates from the inside to the outside of the cantilever while the inside positions (on the x-axis) are positive and the outside positions are negative. Therefore, the x-axis of **Figure S16** is inverted. The current through the nanopore was derived from a line integral along the vertical line in **Figure S14d**, which goes through the pore. The resulting currents are shown in **Figure S16** and indicate that a forward movement results in a current drop first with a subsequent current increase while a backward movement starts with a current increase. We note that the simulation represents a simplified model that is not expected to perfectly fit the current measurements. However, it could be used as a qualitative explanation of the observed differences. As we conducted a 2D-simulation, the current is given in ampere per meter (y-axis).

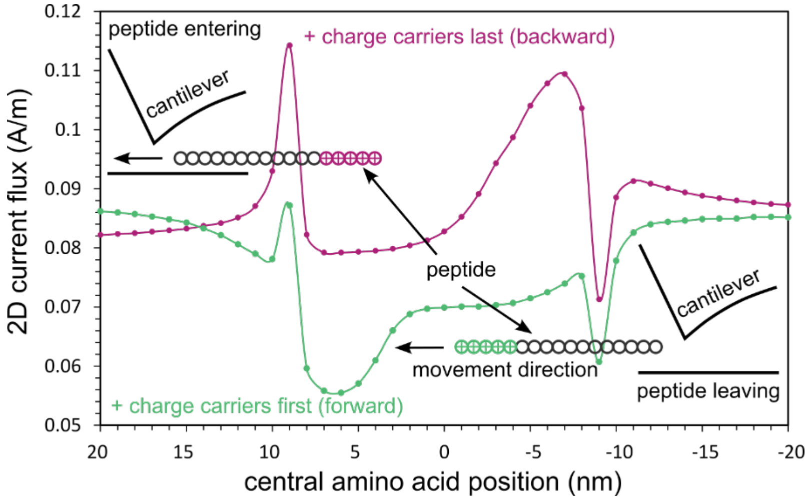

Figure S16: COMSOL simulations of a peptide translocating through a 2 nm small pore confirms orientation-dependent peptide translocation. The purple curve shows a steady-state solution of the peptide movement when the positive amino acids are at the back of the peptide with respect to the movement direction (peptide translocating through N-terminus). The green curve shows the same simulation but when the positive charges of the peptide reach and leave the interface nanopore first (peptide translocating through C-terminus).

S4.7. Current level extraction by change point detection

Figure S17 illustrates an example of the current level extraction. A window with a fixed amount of sample datapoints was shifted over the current signal shifting one data point at a time. For the signal shown in **Fig. 4p-q**, the window's length was chosen to correspond to 5 ms (200 data points). In **Figure S17**, we show a simplified illustration of the method using windows used that contain 5 samples as this is the approximated extent of the electric field. A computed signal with an arbitrary sample rate is shown. Windows that contain a changepoint, a change in the local mean value of the signal, have a higher standard deviation resulting in a peak in the computed standard deviation signal (**Figure S17b**). A peak detection (from Python library scipy.signal module find_peaks (prominence was set to 0.3)) was applied to identify the changepoints. The current level was then calculated by taking the mean in between two changepoints (**Figure S17c**).

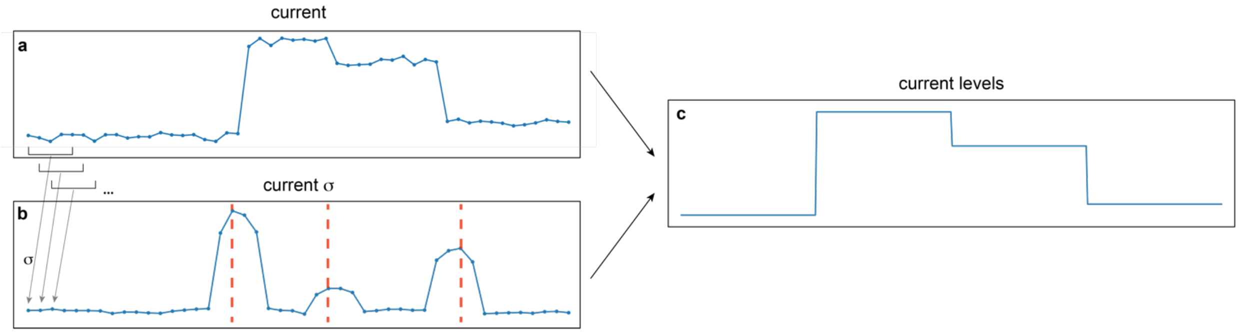

Figure S17: Current level extraction of a computed example current signal. a, The generated raw current signal. **b,** The standard deviation calculated from a rolling window with a length of 5 samples showing peaks at the changepoints. **c,** The current levels extracted from the changepoints and the mean of the current signal in between two successive changepoints.

S4.8. Virtual peptide signal generation

To create a "virtual" peptide signal of a peptide sequence with *n* amino acids (AA1, …, AAn) we considered the different parameters of each amino acid in the sequence, that could have an influence on the current signal upon translocation (*i.e.,* volume, mass, and charge). For each of these parameters, we introduce a weight, *w* to tune the respective influence on the virtual signal. In an ideal case, the observed current signal, *x* would then follow the equation:

$$\begin{pmatrix} AA1_{vol} & AA1_{charge} \\ AA2_{vol} & AA2_{charge} \\ \vdots & \vdots \\ AAn_{vol} & AAn_{charge} \end{pmatrix} \cdot \begin{pmatrix} w_{vol} \\ w_{charge} \end{pmatrix} \propto \begin{pmatrix} x_1 \\ x_2 \\ \vdots \\ x_n \end{pmatrix} \tag{4.7-1}$$

However, the sensitive region of the nanopore typically includes more than one amino acid. Hence, we introduce an averaging operation that, in our case, spans 8 amino acids (arbitrary averaging sizes are possible). Windows of 8 were taken due to the finite extension of the electric field (**Figure S15a**) Thus, the minimal number of amino acids within the sensing volume was assumed to be 8 amino acid lengths, which yields the following equation for the virtual signal:

$$\frac{1}{8}\begin{pmatrix} 1 & 1 & 1 & 1 & 1 & 1 & 1 & 1 & 0 & 0 & \cdots & 0 \\ 0 & 1 & 1 & 1 & 1 & 1 & 1 & 1 & 1 & 0 & \dots & 0 \\ \vdots & \vdots & \vdots & \vdots & \vdots & \vdots & \vdots & \vdots & \vdots & \vdots & \ddots & \vdots \\ 0 & 0 & 0 & \dots & 1 & 1 & 1 & 1 & 1 & 1 & 1 & 1 \end{pmatrix} \cdot \begin{pmatrix} AA1_{vol} & AA1_{charge} \\ AA2_{vol} & AA2_{charge} \\ \vdots & \vdots \\ AAn_{vol} & AAn_{charge} \end{pmatrix} \cdot \begin{pmatrix} w_{vol} \\ w_{charge} \end{pmatrix} \tag{4.7-2}$$

The values used for the specific amino acids of this work are summarized in **Table S3**:

Amino Acid Name	Solution Volume at 25°C (ml/mol)[11]	Surface Charge[12]
Arginine	111.5	+1
Asparagine	74.1	0
Glycine	43.5	0
Isoleucine	107.5	0
Lysine	108.5	+1
Phenylalanine	121.3	0
Serine	60.8	0
Threonine	77.1	0
Tryptophan	144.1	0
Tyrosine	123.6	0

Table S3: Parameters used for the single amino acids in the signal generation. The molecular weights were taken from Sigma Aldrich.

Sliding windows with a length of 10 levels (N amino acids of peptide – averaging window of 8 due to electric field distribution inside the nanopore) were autocorrelated with the measured ionic current signal to find repetitive motifs. If the Pearson correlation factor was higher than a predefined threshold, (0.7) the corresponding part of the signal was marked as a reoccurrence of the respective motif. Overlapping motifs were discarded. The motif with the most reoccurrences with no overlaps is shown in **Fig. S15**. If the reverse representation of the motif exceeded the correlation factor threshold, this reverse reoccurrence was also marked and regarded as a backwards translocation (peptide translocating through C-terminus, **Fig. S15d** as discussed in **Fig. S13**). A virtual signal was then generated as described above, to find correlations between the identified motifs and the translocating peptide. By optimizing the weights (w_{vol} = 0.022 and w_{charge} = -1), a strongly correlating virtual signal with a Pearson correlation factor of 0.95 was found. Using this method, a correlation between the known peptide sequence (Phe peptide) and a highly reoccurring motif within the signal could be found (**Fig. S15e**), which hints at sequence related information in the acquired signal.

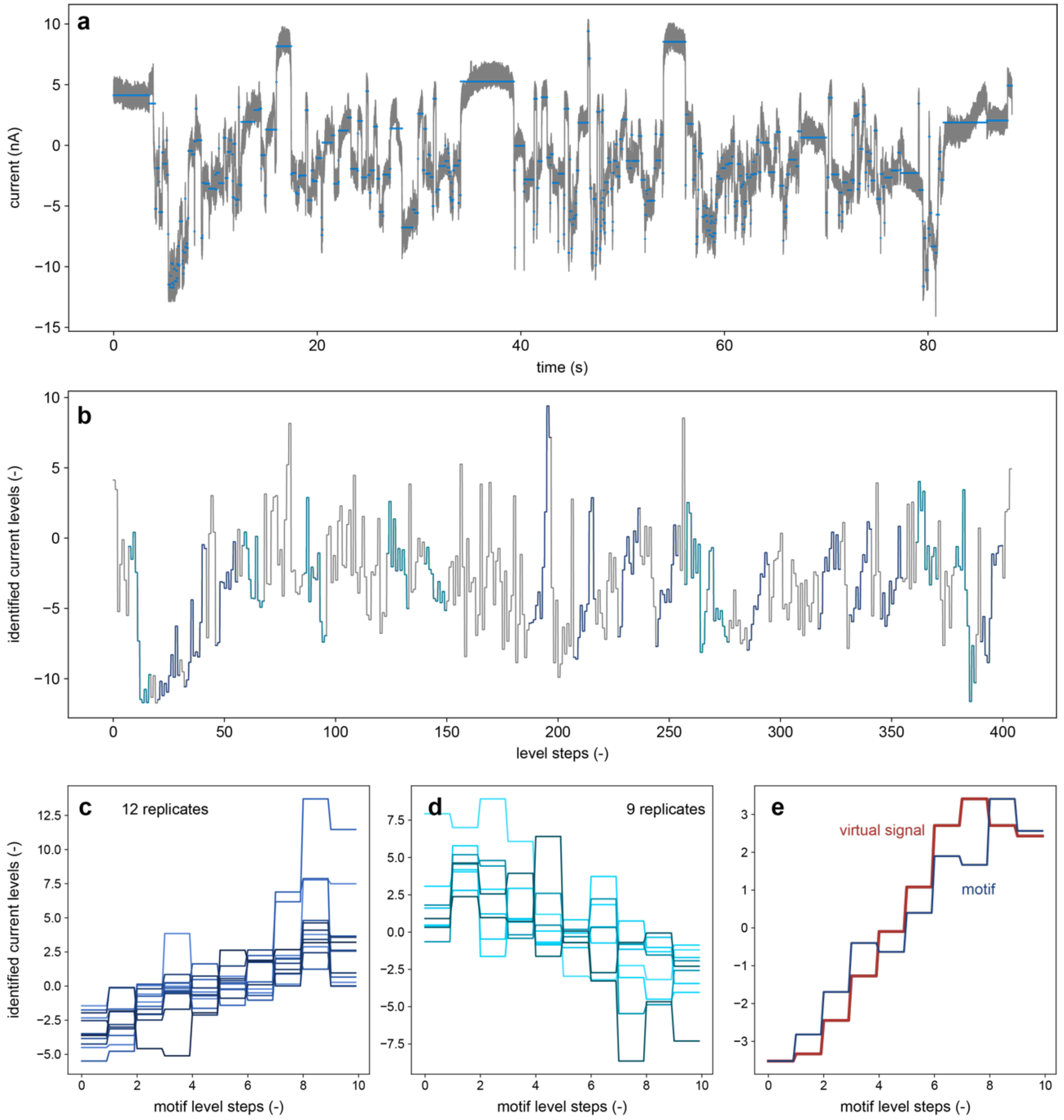

Figure S18: Motif search by autocorrelation of a sliding window and virtual signal from the peptide sequence. a, Current trace of Phe peptide at 0.5 V. **b,** Offline level detection shows distinct current levels. The identified levels are shown with similar length. The mostly reoccurring motif (21 times) is marked in blue both for forward (dark blue) and backward (light blue) reoccurrences. The mean pearson correlation factor of the reoccurring patterns with the main motif was 0.81. Different reoccurrences offset by their median current level for **c,** forward translocations, and **d,** backward translocations). e, An artificial peptide signal was generated by weighting parameters of the single amino acids (mass, charge) as described above. The weights were optimized to yield the largest pearson correlation factor (0.95) of the virtual signal with the main motif. The weights were (w_{vol}, w_{charge}) = (0.022, -1).

S5. Supplementary References

(1) Schlotter, T.; Weaver, S.; Forró, C.; Momotenko, D.; Vörös, J. J.; Zambelli, T.; Aramesh, M. Force-Controlled Formation of Dynamic Nanopores for Single-Biomolecule Sensing and Single-Cell Secretomics. *ACS Nano* **2020**, *14* (10), 12993–13003.

(2) Pavlov, A. N.; Hramov, A. E.; Koronovskii, A. A.; Sitnikova, E. Y.; Makarov, V. A.; Ovchinnikov, A. A. Wavelet Analysis in Neurodynamics. *Physics-Uspekhi* **2012**, *55* (9), 845–875.

(3) Addison, P. S. *The Illustrated Wavelet Transform Handbook: Introductory Theory and Applications in Science, Engineering, Medicine and Finance*; CRC press, **2017**.

(4) Eilers, P. H. C.; Boelens, H. F. M. Baseline Correction with Asymmetric Least Squares Smoothing. *Leiden University Medical Centre Report* **2005**, *1* (1), 5.

(5) Maaten, L. van der; Hinton, G. Visualizing Data Using T-SNE. *J. Mach. Learn. Res.* **2008**, *9*, 2579–2605.

(6) Kobak, D.; Berens, P. The Art of Using T-SNE for Single-Cell Transcriptomics. *Nat. Commun.* **2019**, *10* (1), 1–14.

(7) McInnes, L.; Healy, J.; Melville, J. UMAP: Uniform Manifold Approximation and Projection for Dimension Reduction. *arXiv preprint arXiv:1802.03426* **2018**. https://doi.org/1802.03426. arXiv.

(8) Ester, M.; Kriegel, H.-P.; Sander, J.; Xu, X. A Density-Based Algorithm for Discovering Clusters in Large Spatial Databases with Noise. In *KDD-96 Proceedings*; AAAI: Portland, August 1996, 1996; Vol. 96, pp 226–231.

(9) Ankerst, M.; Breunig, M. M.; Kriegel, H.-P.; Sander, J. OPTICS: Ordering Points to Identify the Clustering Structure. *SIGMOID Rec.* **1999**, *28* (2), 49–60.

(10) Zeng, S.; Wen, C.; Solomon, P.; Zhang, S.-L.; Zhang, Z. Rectification of Protein Translocation in Truncated Pyramidal Nanopores. *Nat. Nanotechnol.* **2019**, *14* (11), 1056–1062.

(11) Counterman, A. E.; Clemmer, D. E. Volumes of Individual Amino Acid Residues in Gas-Phase Peptide Ions. *J. Am. Chem. Soc.* **1999**, *121* (16), 4031–4039.

(12) *PepCalc.com - Peptide property calculator*. https://pepcalc.com/ (accessed 2022-08-24).
